# Synthesis and bioactivity of pyrrole-conjugated phosphopeptides

**DOI:** 10.3762/bjoc.18.17

**Published:** 2022-01-31

**Authors:** Qiuxin Zhang, Weiyi Tan, Bing Xu

**Affiliations:** 1 Department of Chemistry, Brandeis University, 415 South Street, Waltham, MA 02454, USA

**Keywords:** cells, enzyme, N-terminal, peptides, pyrroles, self-assembly

## Abstract

Here we report the synthesis and effect on the cell viability of pyrrole-conjugated phosphopeptides. Encouraged by the selective inhibition of cancer cells by a naphthyl-capped phosphopeptide (Nap-ff_p_y, **1**), we conjugated the heteroaromatic dipyrrole or tripyrrole motif at the N-terminal of short peptides containing phosphotyrosine or phosphoserine and examined the bioactivity of the resulting phosphopeptides (**2**–**10**). Although most of the phosphopeptides exhibit comparable activities with that of **1** against HeLa cells at 200 μM, they, differing from **1**, are largely compatible with HeLa cells at 400 μM. Enzymatic dephosphorylation of **2**–**10**, at 400 μM is unable to induce a dramatic morphological transition of the peptide assemblies observed in the case of **1**. These results suggest that a heteroaromatic motif at the N-terminal of peptides likely disfavors the formation of extensive nanofibers or morphological changes during enzymatic self-assembly, thus provide useful insights for the development of phosphopeptides as substrates of phosphatases for controlling cell fate.

## Introduction

Biomacromolecular assemblies have received considerable attention recently in the field of biomaterials [1–7], among which peptides are of particular interest because of their unique merits, such as ease of design and tailoring (based on the known structures from proteins), good biocompatibility and degradability, and low immunogenicity. For example, recent works have demonstrated the potential of peptide assemblies for a wide range of applications, including drug delivery [8–11], collagen mimic [12], antibacterial [13–14], biomineralization [15–16], mimicry of amyloids [17], cell cultures [18], and tissue engineering [19]. Particularly, the use of enzyme-instructed self-assembly (EISA) [20–21] of peptide assemblies has expanded the applications of peptide assemblies, such as intracellular phase transition [22], molecular imaging [23–33], anisotropic hydrogels [34–35], targeting subcellular organelles [36–40], elimination of pluripotent stem cells (PSC) [41–42], and cancer therapy [43–54]. Being a multistep molecular process to generate non-diffusive peptide assemblies, EISA is a facile and useful approach to explore the emergent properties of peptide assemblies [55–59] in cellular environment. One of the most explored EISA processes is the use of alkaline phosphatase (ALP) to convert the micelles made of phosphopeptides to the nanofibers of peptides via enzymatic dephosphorylation [20,60]. While ALP-catalyzed EISA has received considerable exploration, the structures of the peptide substrates mainly have centered on naphthylacetyl-capped phosphopeptides (e.g., Nap-ff_p_y (**1**)) [36,61–66]. Considering naphthyl is an aromatic group, we decided to explore other aromatic N-terminal capping groups, such as heteroaromatic, N-terminal capping groups of phosphopeptides for EISA because they receive little exploration [67].

Our previous studies have shown nucleobases, as the heteroaromatic groups, are able to act as the N-capping group for EISA of phosphopeptides [67]. In this study, we chose to examine a different type of heteroaromatic group, pyrroles, because pyrrole is adaptable for solid-phase synthesis [68] so that it is feasible to conjugate multiple pyrroles to phosphopeptides. In addition, pyrrole has yet to be incorporated in peptides for EISA, though oligomeric pyrroles have been extensively explored for binding nucleic acids [68]. Based on a naphthyl-capped phosphopeptide (Nap-ff_p_y, **1**), we conjugated heteroaromatic dipyrrole or tripyrrole motifs at the N-terminal of short peptides containing phosphotyrosine or phosphoserine and examined the bioactivity of the resulting phosphopeptides (**2**–**10**). Unexpectedly, enzymatic dephosphorylation of **2**–**10** rarely induces distinct morphological transitions of the peptide assemblies, such as the transition of nanoparticles to nanofibers, which is a transition observed in the case of **1** [43]. Incubating **2**–**10** with HeLa cells reveals that these phosphopeptides exhibit comparable inhibitory activity as that of **1** against the proliferation of HeLa cells at 200 μM. But **2**–**10** at 400 μM, differing drastically from **1**, are largely compatible with HeLa cells, while **1**, at 400 μM, significantly inhibits HeLa cells. These results suggest that morphological transition or extensive self-assembly triggered by enzymatic reactions likely is necessary for the inhibition of HeLa cells by EISA. This work, thus, provides useful insights for the development of phosphopeptide derivatives as enzyme substrates for controlling cell fate.

## Results and Discussion

### Molecular design

**Scheme 1:**
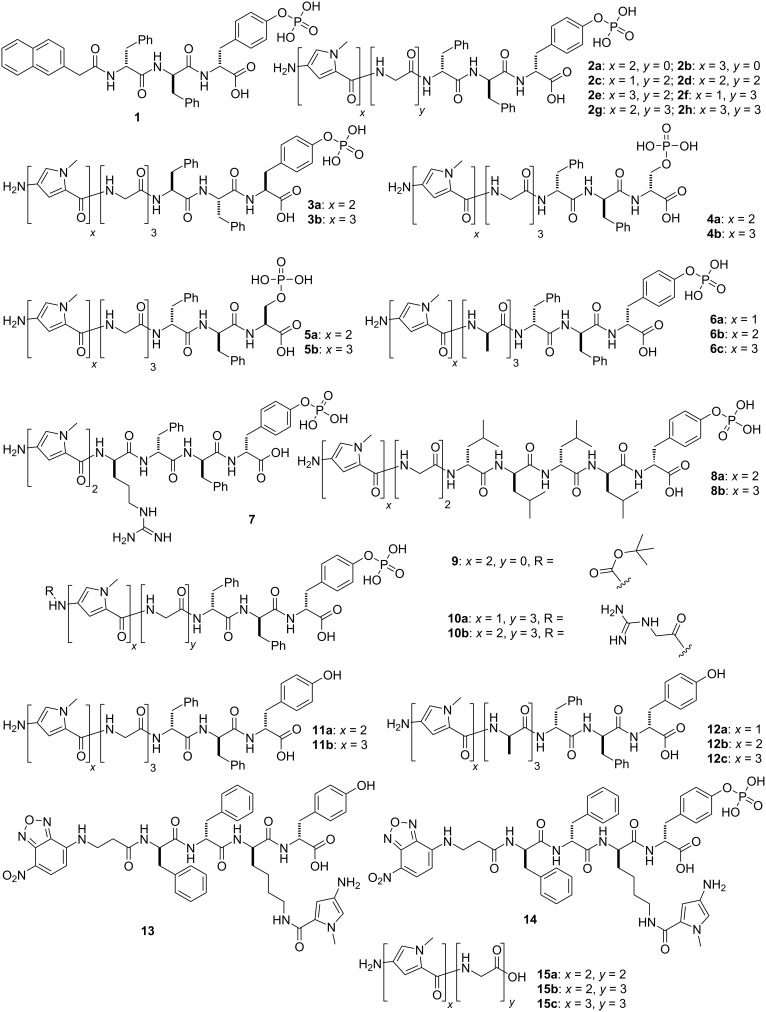
Molecular structures of the parent phosphopeptide **1** and its pyrrole-conjugated analogs **2**–**14**.

As illustrated in Scheme 1, the phosphopeptide (Nap-ff_p_y, **1**) that inhibits HeLa cells consists of three segments, naphthylacetyl (Nap) at the N-terminal of the peptide, a self-assembling motif (ᴅ-phenylalanine-ᴅ-phenylalanine (ff)) [69] as the main backbone, and an enzymatic trigger ᴅ-phosphotyrosine (_p_y). This design presents a substrate of alkaline phosphatase (ALP) for EISA. Based on this structure, a considerable number of studies have investigated the physical properties (enzyme-catalyzed hydrogelation or supramolecular structural transformation) [62,70] and biological properties (cell viability, biostability, and cell-selective growth inhibition) [43,62] of the naphthylacetyl N-terminal-capped phosphopeptides. These studies have produced a variety of phosphopeptides that selectively inhibit cancer cells [38,64–65,71–72]. Encouraged by the results from the naphthylacetyl capped phosphopeptides, we decided to use multiple *N*-methylpyrroles, as the heteroaromatic analog of naphthyl, to cap the N-terminal of phosphopeptides for EISA. According to this rationale, replacing the Nap capping group in **1** with a dipyrrole or a tripyrrole segment at the N-terminal generates **2a** and **2b**. Introducing two or three glycine residues between the pyrrole segment and the self-assembling segment produces **2c**–**h**, which would help to understand the role of a spacer in the molecular structure. As the enantiomers of **2g** and **2h**, respectively, **3a** and **3b** offer an opportunity to assess the effects of stereochemistry of the pyrrole-capped phosphopeptides. Substituting the ᴅ-phosphotyrosine (_p_y) in **2g** and **2h**, by ᴅ-phosphoserine (_p_s), affords **4a** and **4b**, respectively. Switching the ᴅ-phosphoserine in **4a** and **4b** by ʟ-phosphoserine (_p_S), creates **5a** and **5b**. ᴅ-Trialanine replaces triglycine in **2f–h** to produce **6a**–**c**, which should resist to proteases, such as polyglycine hydrolases [73], which are known to cleave at the glycine–glycine site. The addition of an arginine residue in the backbone of **2a** leads to **7**, which bears an additional positive charge compared to **2a**. A ᴅ-tetraleucine (l_4_) replaces ᴅ-diphenylalanine (ff) in **2d** and **2e** to form **8a** and **8b**, respectively. Adding a Boc protecting group at the N-terminus of **2a** produces **9**. Attaching a guanidinoacetic acid motif to **2f** and **2g** results in **10a** and **10b**. **9** and **10** would help test the effects of additional N-terminal modifications. To examine the necessity of enzymatic dephosphorylation, we replaced ᴅ-phosphotyrosine (_p_y) in **2g**, **2h**, **6a**–**c** by ᴅ-tyrosine to yield **11a**, **11b**, **12a**–**c**, respectively. Attaching *N*-methylpyrrole on the sidechain of NBD-ffky or NBD-ffk_p_y [66], another previously studied self-assembling peptide, produces **13** and **14**. In addition, **15a**–**c** consist of only pyrrole and glycine units, which should help delineate the roles of the self-assembling motif and the enzymatic triggers.

### Synthesis

The synthesis of the designed peptides combines solution synthesis of the enzyme trigger or the fluorophore with the solid-phase synthesis of the pyrrole-peptide conjugates. We used phosphorus pentoxide and phosphoric acid to react with ᴅ-tyrosine, ʟ-tyrosine, ʟ-serine, or ᴅ-serine to produce ᴅ-phosphotyrosine, ʟ-phosphotyrosine, ʟ-phosphoserine, or ᴅ-phosphoserine, respectively. After being protected by an Fmoc group, these phosphorylated amino acids are suitable for solid-phase synthesis. We also used solution-phase synthesis to synthesize NBD-β-alanine according to the reported procedures [66]. We used standard Fmoc solid-phase peptide synthesis (SPPS) to generate the peptide segments ((G/GG/GGG)ff_p_y for **2a**–**h**, **9**, **10a**,**b**, GGGFF_p_Y for **3a**,**b**, GGGff_p_s for **4a**,**b**, GGGff_p_S for **5a**,**b**, aaaff_p_y for **6a–c**, rff_p_y for **7**, GGllll_p_y for **8a**,**b**, GGGffy for **11a**,**b**, aaaffy for **12a**–**c**, NBD-ffky for **13**, NBD-ffk_p_y for **14**, GG(G) for **15a**–**c**). Then, we introduced the *N*-methylpyrrole (Py) units into the peptides obtained via solution-phase amide bond formation to produce **2a**–**h**, **3a**,**b**, **4a**,**b**, **5a**,**b**, **6a**–**c**, **7**, **8a**,**b**, **11a**,**b**, **12a**–**c**, **13**, **14**, and **15a**–**c**. We conjugated two Py units successively to the peptide ff_p_y and kept the Boc protecting group of the second Py unit to give a Boc-capped product, **9**. We added guanidinoacetic acid to Py-GGGff_p_y and (Py)_2_-GGGff_p_y for making **10a** and **10b**, respectively. All the products were purified by HPLC.

### Bioactivity

**Figure 1:**
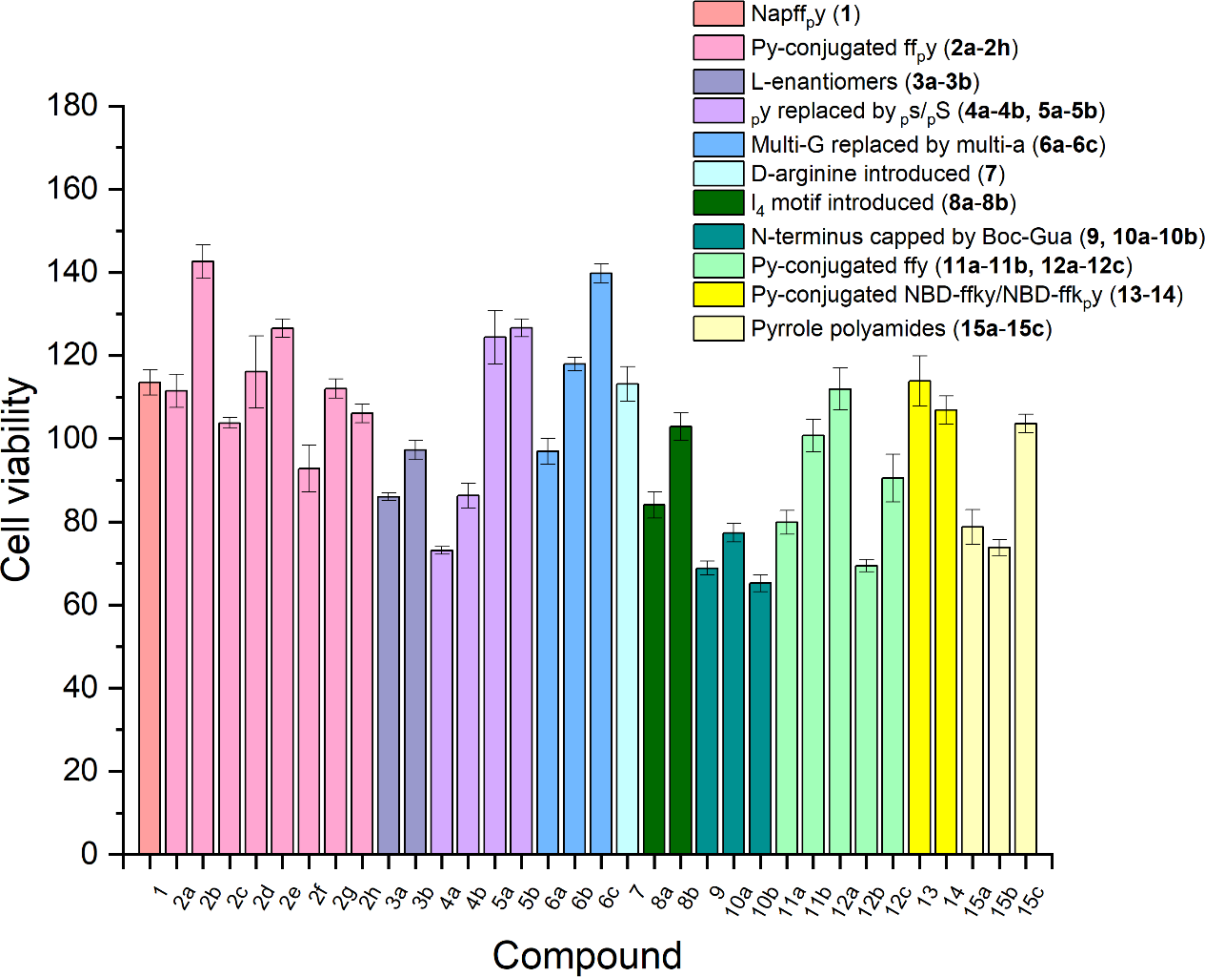
Cell viability of HeLa cells treated with 200 μM of each compound for 24 h.

We examined the cytotoxicity of the synthesized compounds by incubating them with HeLa cells because HeLa cells overexpress ALP and are widely available. By incubating 200 μM of each compound with HeLa cells for 24 hours, we employed MTT assay to determine the cell viability of the HeLa cells. As shown in Figure 1, the MTT results indicate that most of the pyrrole-conjugated phosphopeptides, at 200 μM, are comparable with the activity of **1** against the HeLa cells. In terms of the pyrrole-conjugated ff_p_y series (**2a**–**h**), all of the compounds are rather compatible with the cells, with the cell viability ranging from 92.8% (**2f**) to 142.6% (**2b**). The incubation of the ʟ-enantiomers **3a** and **3b** with HeLa cells results in cell viabilities of 86.0% and 97.2%, respectively. While ʟ-phosphoserine peptides, **5a** and **5b**, hardly inhibit the HeLa cells, ᴅ-phosphoserine peptides, **4a** and **4b**, moderately inhibit the proliferation of the HeLa cells, among which **4a** decreases cell viability to 69.2%. **6a**–**c**, containing a ᴅ-trialanine motif and one to three pyrrole motifs at the N-terminal, hardly inhibit the HeLa cells. Compound **7**, formed by the introduction of a ᴅ-arginine residue into **2a**, exhibits similar cytotoxicity as that of **2a**. **8a** and **8b**, formed by replacing the ff motif in **2d** and **2e** with l_4_, exhibit slight and little cytotoxicity, respectively. Capping the N-terminus with a Boc-protecting group (**9**) or a guanidinoacetic acid motif (**10a** and **10b**) renders the molecules with higher cytotoxicity (resulting in cell viabilities of about 70%) than those of **2f** and **2g**. Compounds without phosphorylation (**11a**, **11b** and **12a**–**c**) show similar activity compared to their phosphorylated counterparts. These results indicate that these peptide assemblies are rather compatible with cells. Compounds **13** and **14**, both possessing a pyrrole building block on the side chain, hardly inhibit the HeLa cells. While **15a** and **15b** exhibit slight cytotoxicity, **15c** is less cytotoxic. This result agrees with the observation that dipyrrole-conjugated peptides, in general, exhibit higher inhibitory activity against HeLa cells than mono- or tripyrrole-conjugated peptides do. It is interesting that **4a** exhibits higher inhibitory activity than that of **2g**, despite that **4a** bears ᴅ-phosphoserine and **2g** bears ᴅ-phosphotyrosine. This result likely warrants further investigation on **4a**. Another dipyrrole-conjugated peptide, **6b**, was also selected for further study as a comparison to **4a** since it shows good cell compatibility at 200 μM.

**Figure 2:**
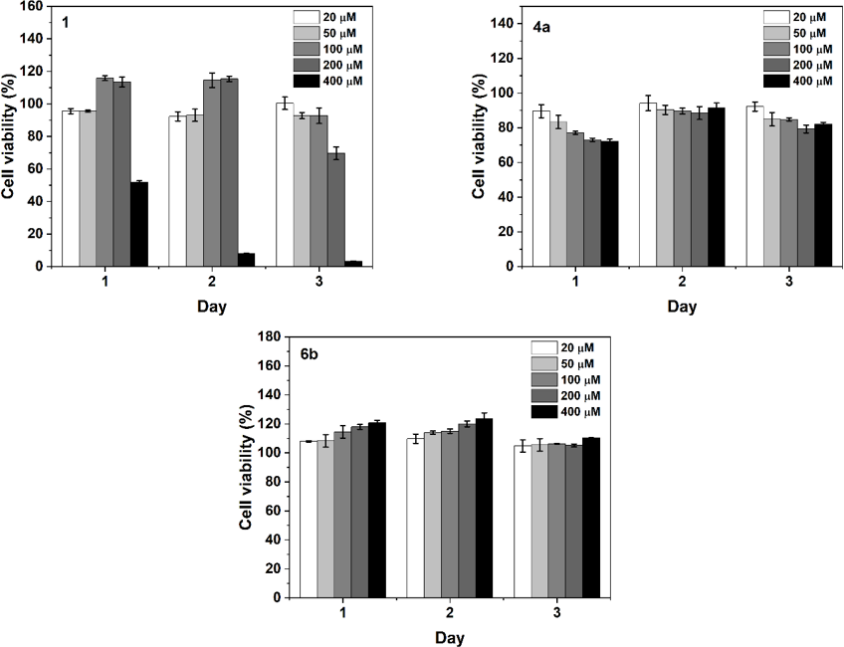
Cell viability of HeLa cells treated with 20 μM, 50 μM, 100 μM, 200 μM and 400 μM of **1**, **4a** and **6b** for 1, 2, and 3 days.

We also compared the cell viability of HeLa cells incubated with **1**, **4a**, and **6b** at different concentrations for 3 days. As shown in Figure 2, the three compounds result in different cell viabilities versus concentration and incubation time. **1** hardly inhibits the proliferation of the cells at concentrations lower than 100 μM over the 3 days, while 200 μM of **1** begins to reduce cell viability on day 3. At the concentration of 400 μM, **1** drastically decreases the cell viability to 52.0% on day 1, while longer incubation time further inhibits cell proliferation, resulting in a cell viability of only 3.4% on day 3. This result agrees well with previous results [43,62]. Unlike **1**, **4a** slightly inhibits cell proliferation, with cell viability ranging from 89.6% to 72.3% with increasing concentrations from 20 to 400 μM. However, the inhibitory effect of **4a** largely vanishes on day 2 and 3 of the incubation. This result implies that cells likely are able to degrade **4a** overtime. Unlike **1** and **4a**, **6b**, exhibiting little cytotoxicity with increasing compound concentration or incubation time, is rather compatible to the cells. These results indicate that the replacement of Nap by pyrroles largely results in cell compatible phosphopeptide derivatives.

### Enzymatic conversion

**Figure 3:**
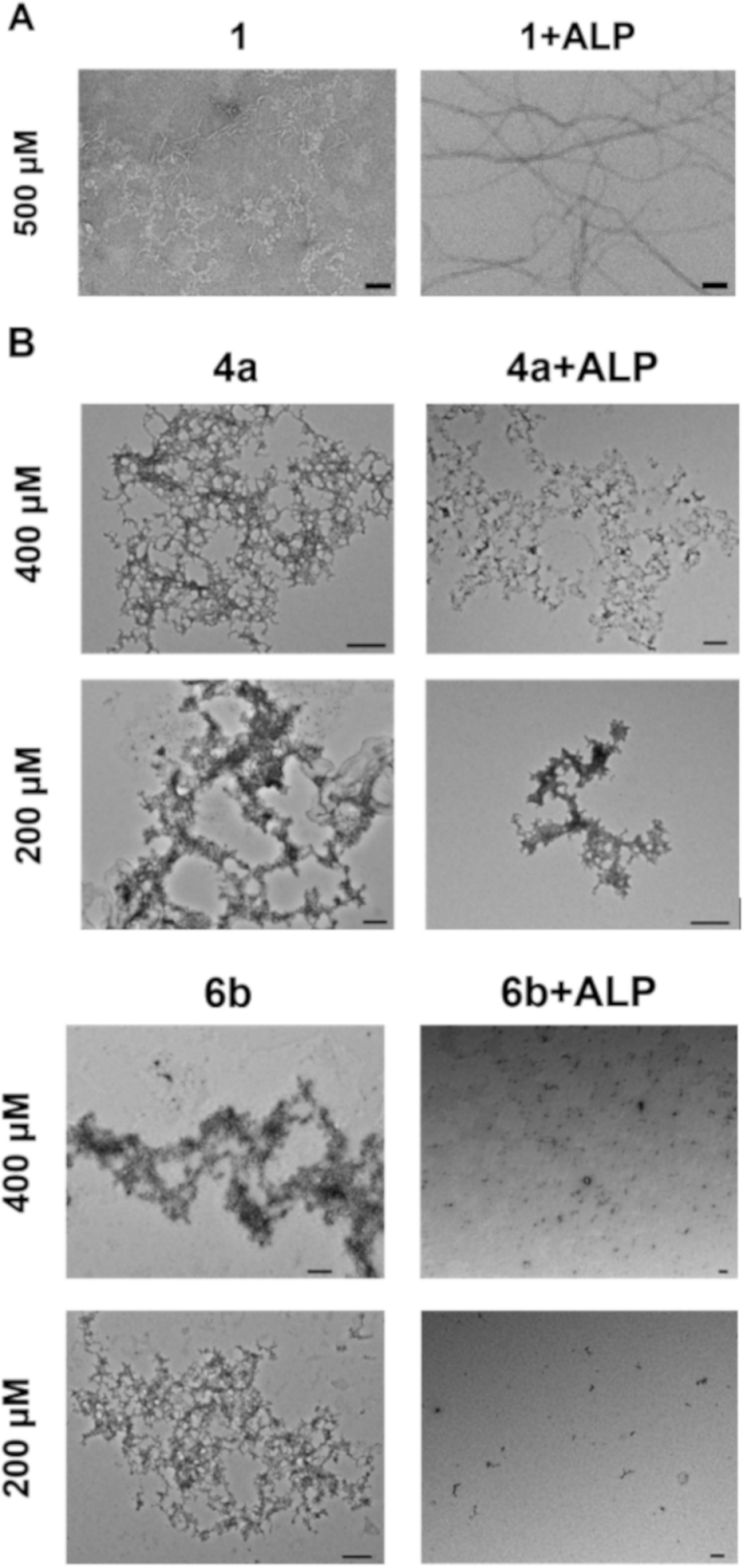
A) TEM images of **1** before and after addition of ALP (0.5 U/mL) in PBS buffer (pH 7.4). Scale bar is 100 nm. Figure 3A was reprinted with permission from [62] https://pubs.acs.org/doi/10.1021/bm5010355, Copyright 2014 American Chemical Society. This content is not subjected to CC-BY 4.0. Permission requests related to the material excerpted should be directed to the ACS. B) TEM images of representative pyrrole-conjugated peptides **4a** and **6b** before and after the addition of ALP (1 U/mL) in PBS buffer (pH 7.4). Scale bar is 200 nm.

To further investigate the reason for the different activities in cell assays among the three compounds, **1**, **4a** and **6b**, we employed transmission electron microscopy (TEM) to study the morphologies of the assemblies of these compounds before and after enzymatic dephosphorylation. Previous literature has demonstrated that **1**, as a precursor, is capable of forming hydrogels upon dephosphorylation catalyzed by ALP [43,62]. To be more specific, ALP enables a morphological transition from nanoparticles of **1** to networks of nanofibers of the corresponding dephosphorylated peptides, resulting in hydrogelation (Figure 3A) [62]. In comparison with **1**, **4a**, forming networks of nanoparticles at 400 μM and 200 μM, exhibits little change in the morphology of the assemblies after being incubated with 1 U/mL of ALP for 24 h (Figure 3B), although LC–MS confirms the enzymatic dephosphorylation of **4a** (Supporting Information File 1, Figure S32). Similarly, **6b** also forms dense nanoparticulate networks at 400 μM and 200 μM (Figure 3B). After the addition of 1 U/mL of ALP for 24 h, the nanoparticles remain, although the networks dissociate. Similarly, LC–MS confirms the enzymatic dephosphorylation of **6b** (Supporting Information File 1, Figure S33) after the addition of ALP. In addition to the TEM images, we used circular dichroism (CD) to reveal the secondary structures of the assemblies before and after enzymatic action. The spectra show a significant change in the signal upon ALP treatment for **1**, but merely slight changes for **4a** and **6b**, which are consistent with the TEM images (Supporting Information File 1, Figure S34). The differences in the morphological transition between **1** and **4a** or **6b** revealed by TEM and CD suggest that the shape-shifting (from nanoparticles to nanofibers) likely contributes to the cell death caused by **1** over days 2 and 3. These results together indicate the drastic morphological changes of phosphopeptides upon the addition of ALP may serve as a useful indicator to predict the cell compatibility or cytotoxicity of phosphopeptides.

## Conclusion

In summary, this work reports the design and synthesis of a series of pyrrole-conjugated phosphopeptides. These pyrrole-conjugated phosphopeptides are largely cell compatible and are unable to undergo drastic morphological transitions, such as from nanoparticles to nanofibers. These results, contrary to the result obtained from **1**, suggest that the morphological transition of the peptide assemblies resulted from enzymatic reaction likely is responsible for disrupting cellular processes to inhibit cells. Other factors, such as endocytosis of the phosphopeptides [74] and the cellular localization of the peptide assemblies [75], certainly could contribute to the cell compatibilities of the pyrrole-conjugated phosphopeptides, which remain to be determined.

## Supporting Information

File 1 Experiment part.
