# Supplementary material for: Synthesis and bioactivity of pyrrole-conjugated phosphopeptides
Source: Beilstein J Org Chem. 2022 Jan 31;18:159–66. doi: 10.3762/bjoc.18.17 (PMC8822458; doi:10.3762/bjoc.18.17)
Supplement: File 1 — Experiment part. [file Beilstein_J_Org_Chem-18-159-s001.pdf]

BEILSTEIN JOURNAL OF ORGANIC CHEMISTRY

Supporting Information

for

Synthesis and bioactivity of pyrrole-conjugated phosphopeptides

Qiuxin Zhang, Weiyi Tan and Bing Xu

Beilstein J. Org. Chem. **2022,** *18,* 159–166. doi:10.3762/bjoc.18.17

Experiment part

License and Terms: This is a supporting information file under the terms of the Creative Commons Attribution License (https://creativecommons.org/licenses/by/4.0). Please note that the reuse, redistribution and reproduction in particular requires that the author(s) and source are credited and that individual graphics may be subject to special legal provisions.
The license is subject to the *Beilstein Journal of Organic Chemistry* terms and conditions: (https://www.beilstein-journals.org/bjoc/terms)

Table of contents

S1. Materials and instruments

S2. Synthesis and characterization of compounds

S3. Cell culture and cell viability

S4. TEM sample preparation

S5. LC-MS

S1. Materials and instruments

2-Cl-trityl chloride resin (1.0–1.2 mmol/g), HOBt, Fmoc-OSu and Fmoc-protected amino acids were purchased from GL Biochem (Shanghai, China), HBTU from Chem-Impex International (Wood Dale, IL), and Boc-Py-OBt and guanidinoacetic acid from Sigma-Aldrich. Other chemical reagents and solvents were purchased from Fisher Scientific. Alkaline phosphatase was purchased from Biomatik (Cat. No. A1130, alkaline phosphatase [ALP], >1300U/mg, in 50% glycerol). All the chemicals and solvents were used as received from commercial sources without further purification. Minimum Essential Media (MEM), fetal bovine serum (FBS) and penicillin-streptomycin were purchased from Gibco by Life Technologies. All compounds were purified using a reverse phase HPLC (Agilent 1100 Series) with an X-Terra C18 RP column, and HPLC grade water (0.1% TFA) and HPLC grade acetonitrile (0.1% TFA) were used as eluents. LC-MS spectra were obtained with a Waters Acquity Ultra Performance LC with Waters MICROMASS detector. Transmission electron microscopy (TEM) images were obtained with a Morgagni 268 transmission electron microscope. Circular dichroism (CD) spectra were obtained with a Jasco J-810 Spectropolarimeter.

S2. Synthesis and characterization of compounds

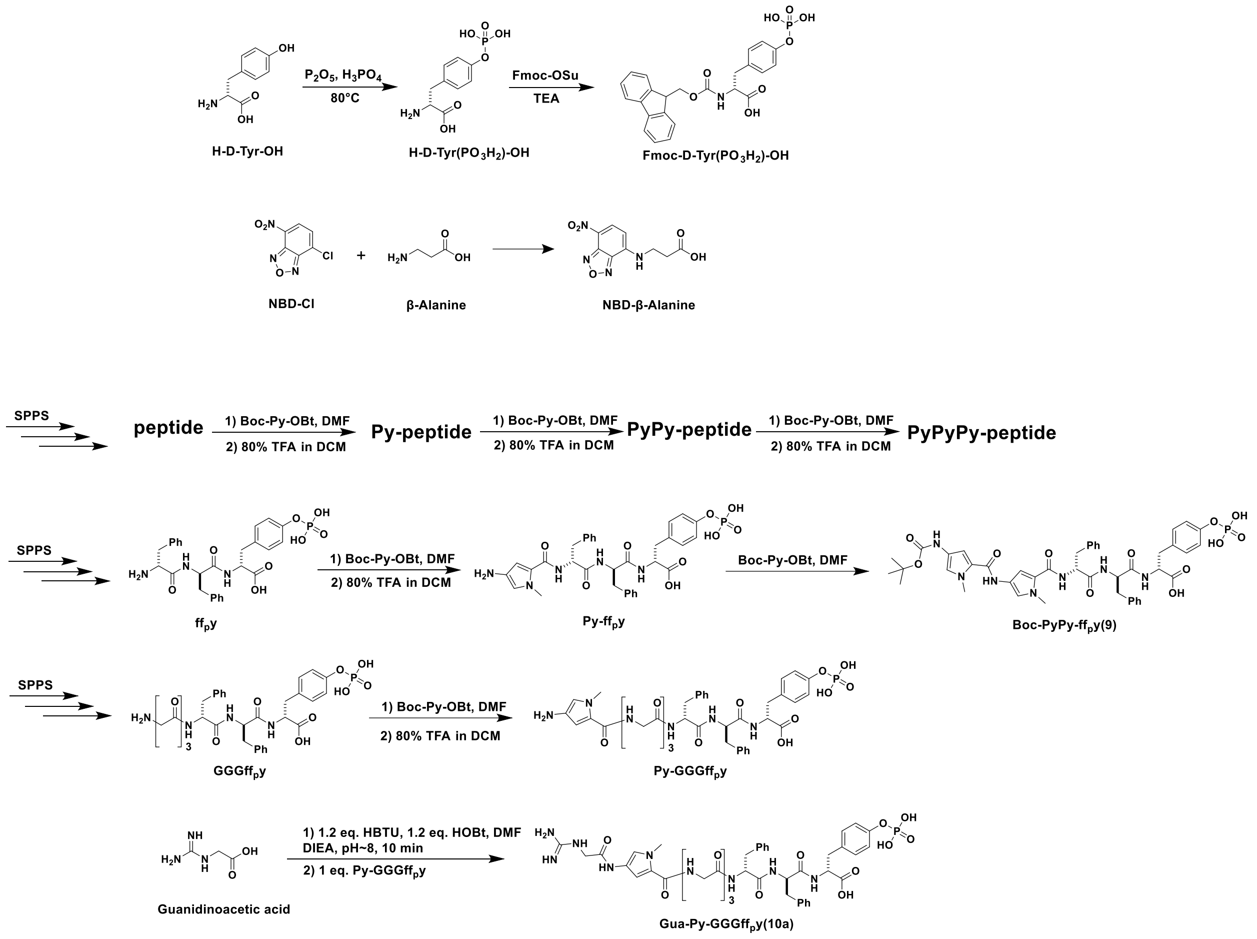

Scheme S1. Synthesis route of compounds.

Synthesis of Fmoc-D-Tyr(PO_3H_2)-OH

Fresh phosphorus pentoxide (35 mmol, 10.0 g) and phosphoric acid (133 mmol, 13.0 g) were added to a round-bottom flask under the protection of nitrogen gas, followed by the addition of D-tyrosine (18 mmol, 3.22 g) to the flask under stirring. The reaction mixture was stirred and heated for 24 h at 80 °C. Upon the addition of 30 mL of water to the mixture, the stirring proceeded for another 30 min at 80 °C. The reaction mixture was then cooled to room temperature and added to butanol (650 mL) dropwise and recrystallized

at 4 °C overnight. White precipitate was collected via filtration and washed with iced water, ethanol and ether in sequence. The final white powder (H-D-Tyr(PO_3H_2)-OH) was collected for the next step.

In order to introduce the Fmoc group, H-D-Tyr(PO_3H_2)-OH (2 mmol, 522 mg) obtained from the previous step was dissolved in 20 mL of water, and Fmoc-OSu (2.4 mmol, 808 mg) dissolved in 20 mL of acetonitrile was added. After adjusting the final pH to ~8 using triethylamine, the reaction mixture was stirred for 1.5 h at room temperature. Upon the removal of acetonitrile by evaporation, 60 mL of water was added and 1 M HCl was used to acidify the solution to pH ~3. The solution was then extracted by ethyl acetate for 3 times, followed by washing of the organic phase with 1 M HCl, water and brine. The solution was dried on Na_2SO_4, filtered and concentrated by evaporation. The final product Fmoc-D-Tyr(PO_3H_2)-OH was collected as a white powder.

Fmoc-L-Tyr(PO_3H_2)-OH was synthesized following the same procedure except for the use of L-tyrosine instead of D-tyrosine.

Fmoc-L-Ser(PO_3H_2)-OH and Fmoc-D-Ser(PO_3H_2)-OH were synthesized following the same procedure except for the use of 1.89 g (18 mmol) of L-serine and D-serine respectively instead of D-tyrosine.

Synthesis of NBD-β-alanine

β-alanine (5 mmol, 445 mg) and potassium carbonate (15 mmol, 2g) were dissolved in 15 mL of water, to which the solution of NBD-Cl (5 mmol, 1g) in 30 mL of methanol was added dropwise under the protection of nitrogen gas. The reaction was stirred at room

temperature for 3 h, followed by the removal of methanol by evaporation. 70 mL of water was added and 1 M HCl was used to acidify the solution to pH ~3. The solution was then extracted by diethyl ether for 3 times, and the organic phase was dried on Na_2SO_4, filtered and concentrated by evaporation. The final product NBD-β-alanine was collected as a dark yellow powder.

Peptide synthesis

Standard Fmoc solid phase peptide synthesis was followed when synthesizing the peptide segments ((G/GG/GGG)ff$_p$y for **2a–h**, **9**, **10a**,**b**, GGGFF$_p$Y for **3a**,**b**, GGGff$_p$s for **4a**,**b**, GGGff$_p$S for **5a**,**b**, aaaff$_p$y for **6a–c**, rff$_p$y for **7**, GGllll$_p$y for **8a**,**b**, GGGffy for **11a**,**b**, aaaffy for **12a–c**, NBD-ffky for **13**, NBD-ffk$_p$y for **14**, GG(G) for **15a–c**) [1]. For each of the peptide segments, 2-chlorotrityl chloride resin and necessary Fmoc-protected amino acids with proper side-chain protecting groups were used. The first amino acid was added to the swollen resin at a ratio of about 0.6 mmol/g of resin. Capping reagent (DCM/MeOH/DIEA = 17:2:1) was used to protect the remaining active sites on the resin after loading the first amino acid. To cleave the Fmoc group, 20% piperidine in DMF was used. HBTU and HOBt were used as coupling reagents to assist the addition of the next Fmoc-protected amino acid to the free amino group of the first one. The crude peptides were cleaved from the resin using 95% TFA, 2.5% TIS and 2.5% H_2O for 1 h. Upon removal of the solvent by evaporation, the crude products were collected for further purification by reverse-phase HPLC.

Synthesis of 2a–h, 3a,b, 4a,b, 5a,b, 6a–c, 7, 8a,b, 11a,b, 12a–c, 13, 14, 15a–c

N-methyl pyrrole (Py) units were introduced into the peptides obtained from the previous steps via liquid phase peptide synthesis. 1 equivalent of corresponding peptide (final concentration ~0.018M) and 1.2 equivalent of 4-(Boc-amino)-1-methylpyrrole-2-carboxylic acid 1,2,3-benzotriazol-1-yl-ester (Boc-Py-OBt) (final concentration ~0.022M) were dissolved in 2 mL of dry *N*,*N*-dimethylformamide (DMF). *N*-diisopropylethylamine (DIEA) was added to adjust the pH of the solution to ~8. After being stirred at room temperature overnight, the solvent was removed by evaporation. The Boc protecting group was then cleaved using 80% trifluoroacetic acid (TFA) in dichloromethane (DCM) at room temperature for 1 h, and the solvent was again removed by evaporation. The second and third Py unit were conjugated to the free amino group of the previous one following the same procedure. Finally, the crude products were collected for further purification by reverse-phase HPLC.

Synthesis of 9

9 was synthesized by conjugating two Py units successively to the peptide $ff_{p}y$ following the previous procedure, while the Boc protecting group of the second Py unit was reserved on the backbone to give a Boc-capped product. After the removal of the solvent by evaporation, the crude product was collected for further purification by reverse-phase HPLC.

Synthesis of 10a,b

10a and **10b** were first synthesized following previous procedures to obtain Py(Py)-GGGff$_p$y. Afterwards, 1.2 equivalent of guanidinoacetic acid (final concentration ~0.019 M), 1.2 equivalent of HBTU (final concentration ~0.019 M) and 1.2 equivalent of HOBt (final concentration ~0.019 M) were added to 2 mL of dry DMF. After adjusting the pH to ~8 using DIEA, the reaction mixture was stirred at room temperature for 10 min. 1 equivalent of corresponding peptide (final concentration ~0.016 M) was then added to the activated guanidinoacetic acid. The reaction mixture was stirred overnight. After the removal of the solvent by evaporation, the crude product was collected for further purification by reverse-phase HPLC.

S3. Cell culture and cell viability

HeLa cell line was purchased from American Type Culture Collection (ATCC, Manassas, VA, USA). HeLa cells were cultured in MEM medium with 100 U mL^{-1} penicillin and 100 μg mL^{-1} streptomycin. Cells were cultured at 37 °C in a humidified atmosphere of 5% CO_2. MTT assay was used to determine the cytotoxicity of the compounds against HeLa cells. Cells were seeded in 96-well plate at 1.0×10^5 cells/well for 24 hours followed by culture medium removal. Fresh culture medium containing different concentrations of compounds were then added. After incubated at 37 °C for 24/48/72 hours, the culture medium with compounds were replaced by fresh culture medium plus 10 μL of MTT solution (5 mg/mL). After incubated at 37 °C for another 4 hours, 100 μL of SDS-HCl solution was added to stop the reduction and dissolve formazan formed. A DTX880

Multimode Detector was used to measure the absorbance of each well at 595 nm, and cell viability was calculated as percentage relative to untreated cells. Data were obtained from three independent wells (n = 3).

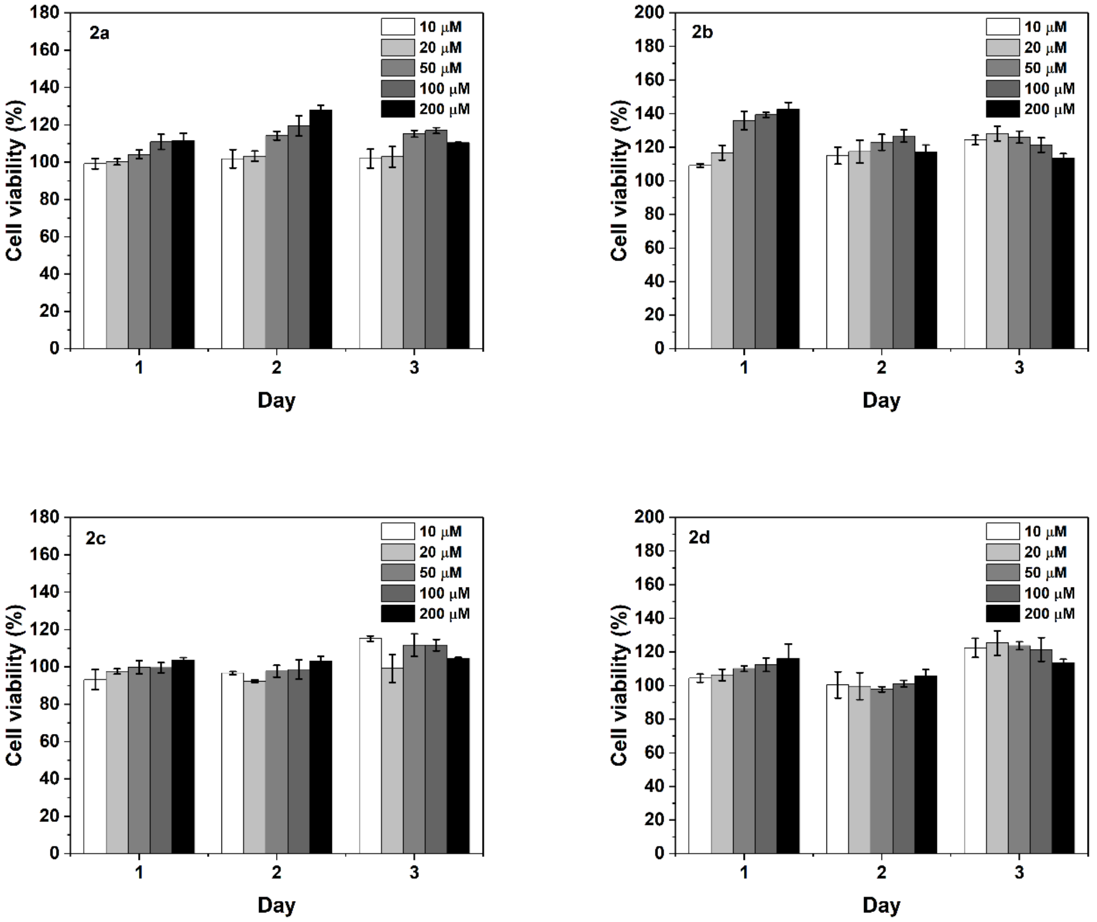

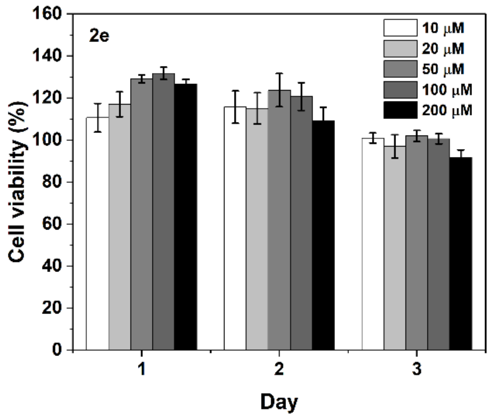
2e
10 μM
20 μM
50 μM
100 μM
200 μM
Cell viability (%)
0
20
40
60
80
100
120
140
160
1
2
3
Day

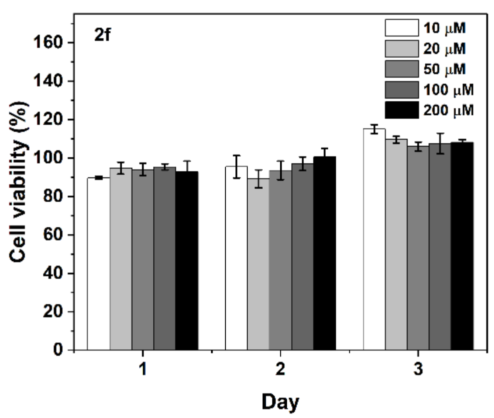
2f
10 μM
20 μM
50 μM
100 μM
200 μM
Cell viability (%)
0
20
40
60
80
100
120
140
160
1
2
3
Day

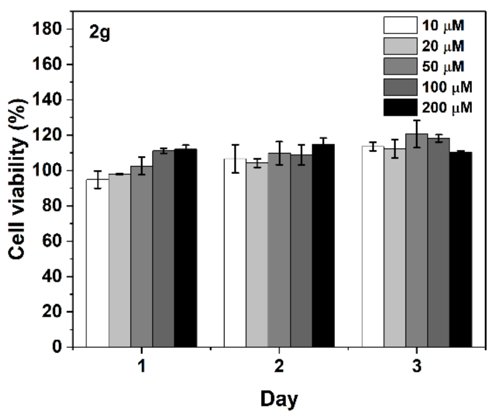
2g
10 μM
20 μM
50 μM
100 μM
200 μM
Cell viability (%)
0
20
40
60
80
100
120
140
160
180
1
2
3
Day

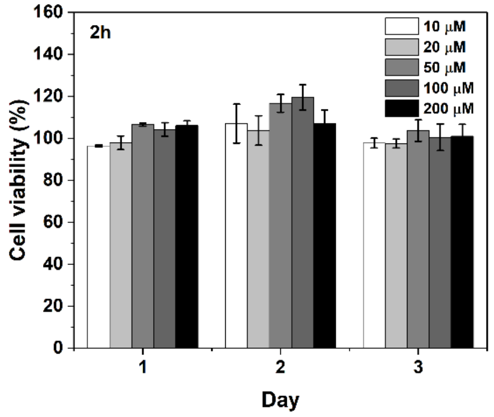
2h
10 μM
20 μM
50 μM
100 μM
200 μM
Cell viability (%)
0
20
40
60
80
100
120
140
160
1
2
3
Day

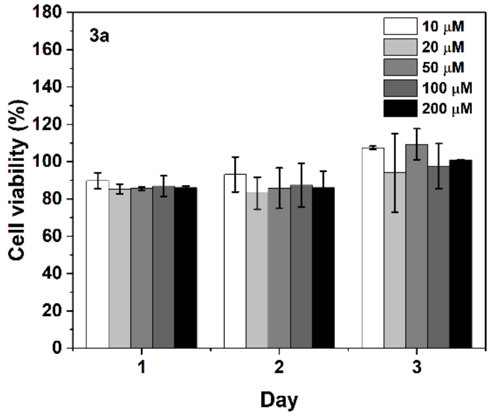
3a
10 μM
20 μM
50 μM
100 μM
200 μM
Cell viability (%)
0
20
40
60
80
100
120
140
160
180
1
2
3
Day

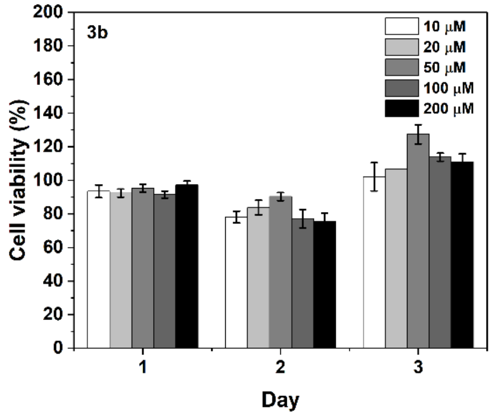
3b
10 μM
20 μM
50 μM
100 μM
200 μM
Cell viability (%)
0
20
40
60
80
100
120
140
160
180
200
1
2
3
Day

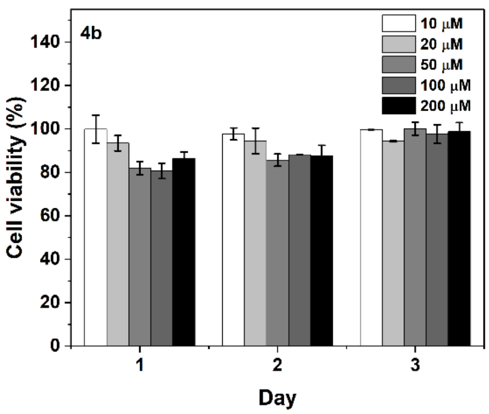
4b
10 μM
20 μM
50 μM
100 μM
200 μM
Cell viability (%)
Day

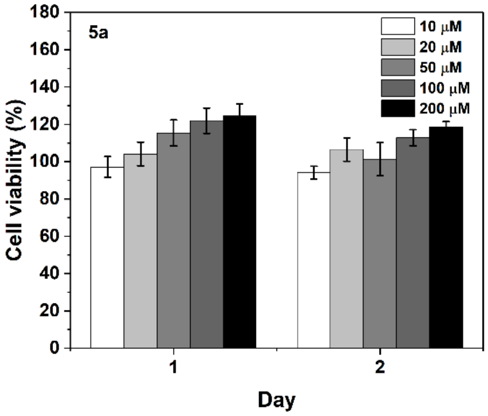
5a
10 μM
20 μM
50 μM
100 μM
200 μM
Cell viability (%)
Day

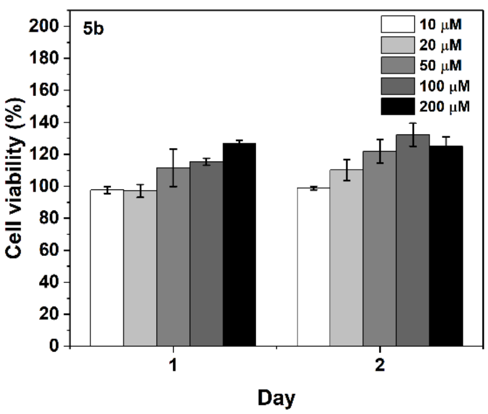
5b
10 μM
20 μM
50 μM
100 μM
200 μM
Cell viability (%)
Day

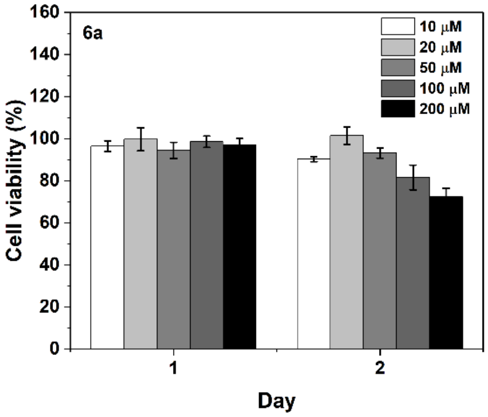
6a
10 μM
20 μM
50 μM
100 μM
200 μM
Cell viability (%)
Day

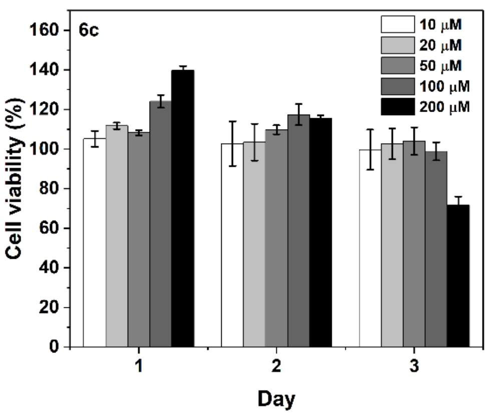
6c
10 μM
20 μM
50 μM
100 μM
200 μM
Cell viability (%)
Day

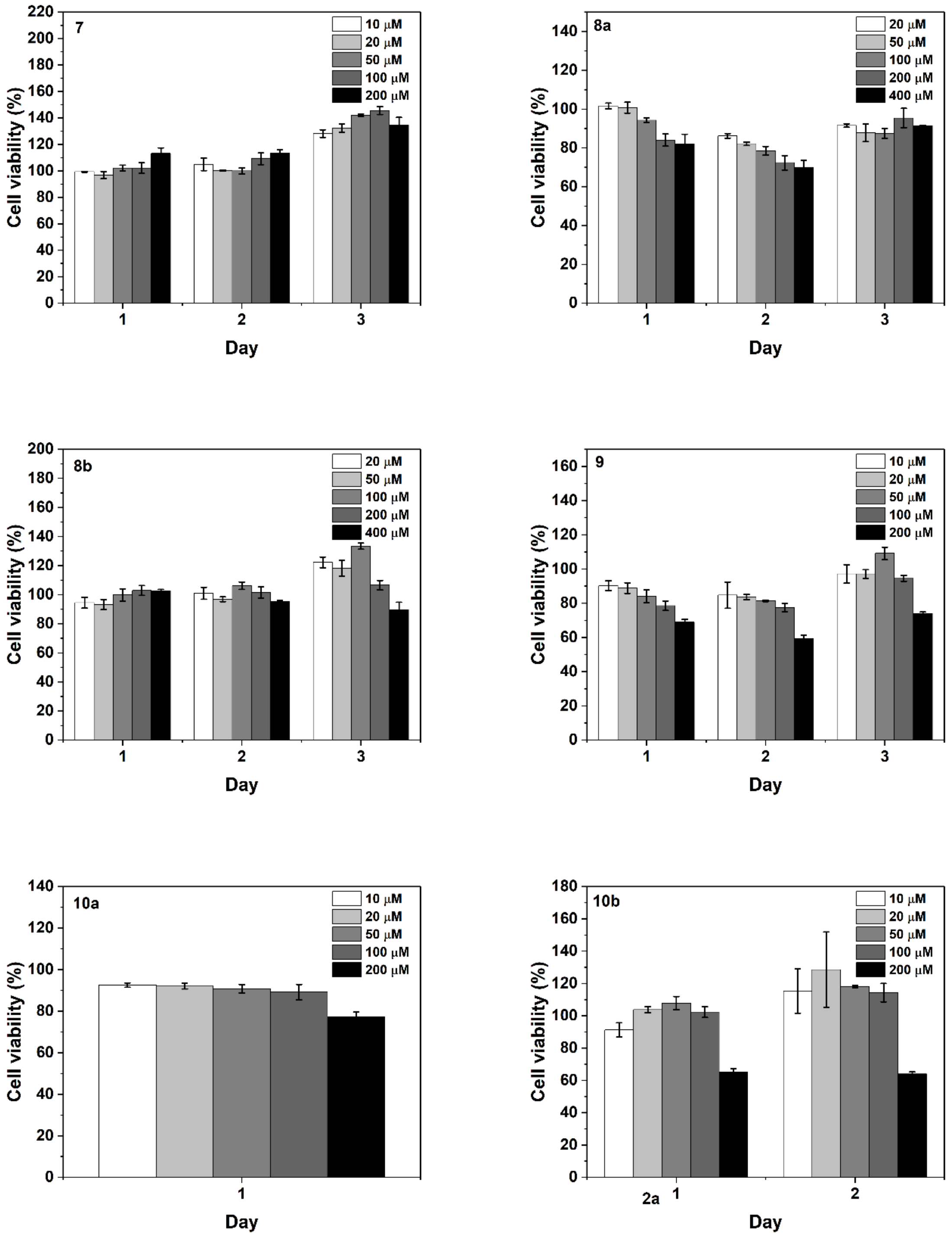
7
8a
8b
9
10a
10b
2a
Cell viability (%)
Day
10 µM
20 µM
50 µM
100 µM
200 µM
400 µM

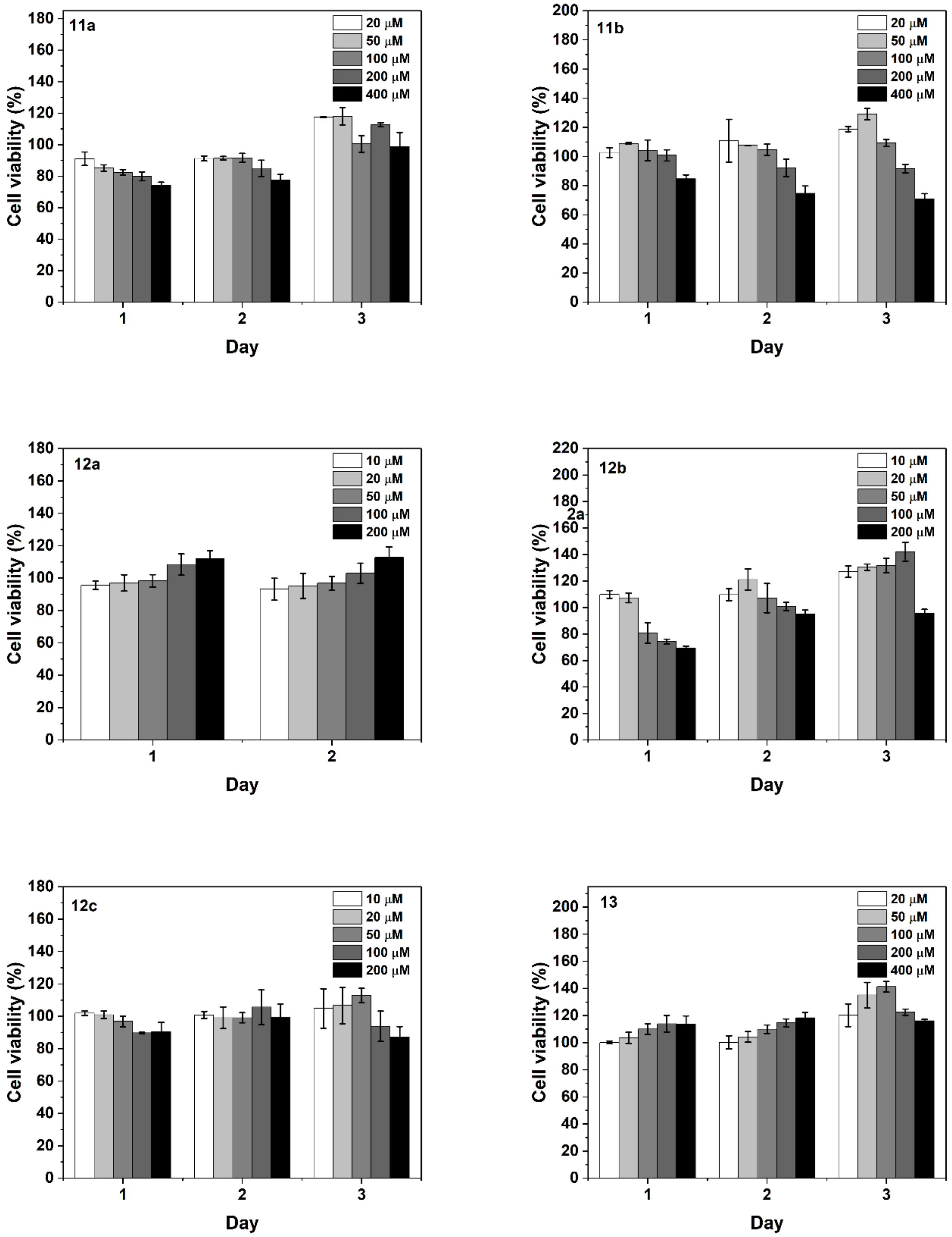
11a
11b
12a
12b
12c
13
Cell viability (%)
Day
20 μM
50 μM
100 μM
200 μM
400 μM
10 μM
2a

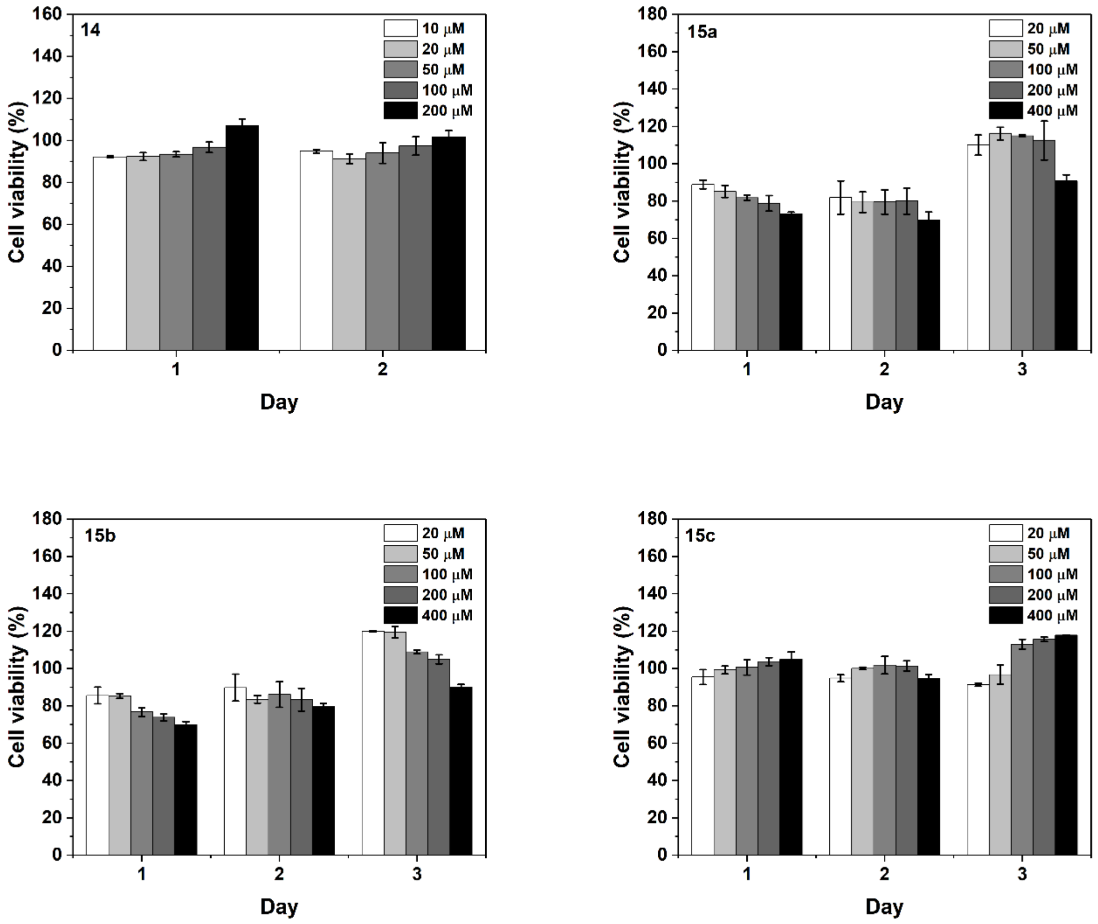

Figure S1. Cell viability of HeLa cells treated with different compounds.

S4. TEM sample preparation

400-mesh copper grids coated with continuous thick carbon film (~35 nm) were glow discharged, on which 3 μL of samples were placed. After washed with ddH_2O and uranyl acetate (UA), the sample-loaded grids were stained with UA. The residual UA was removed by filter paper and then air-dried.

S5. LC-MS

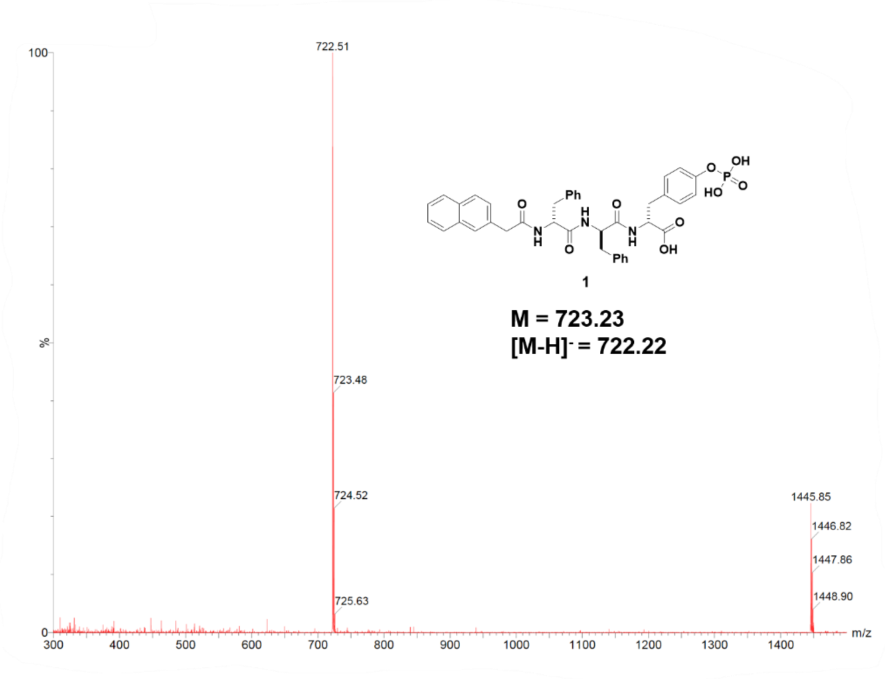

Figure S2. Mass spectrum of **1** (*m/z* = 722.51).

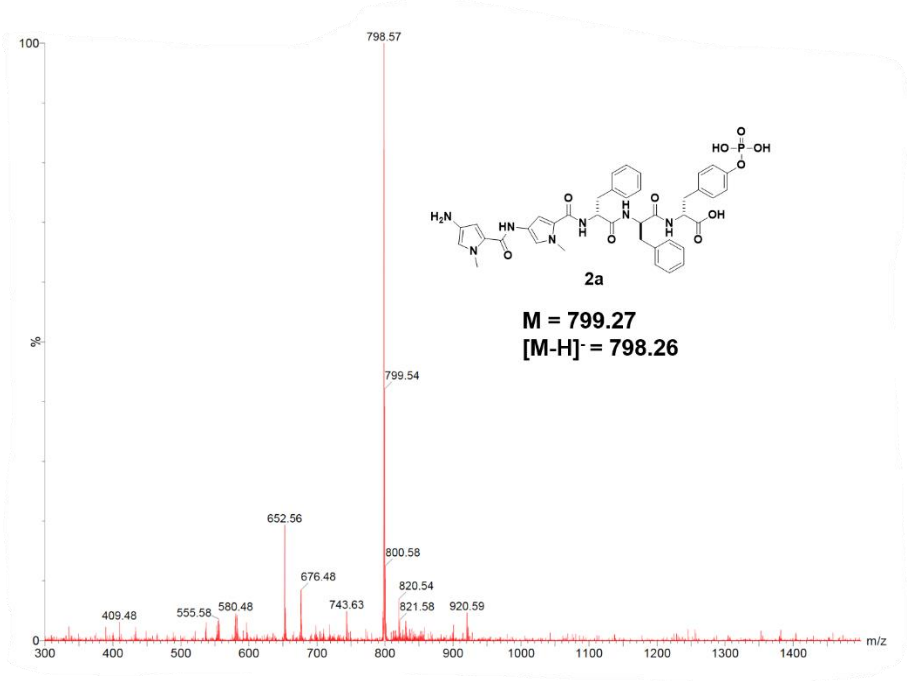

Figure S3. Mass spectrum of **2a** (*m/z* = 798.57).

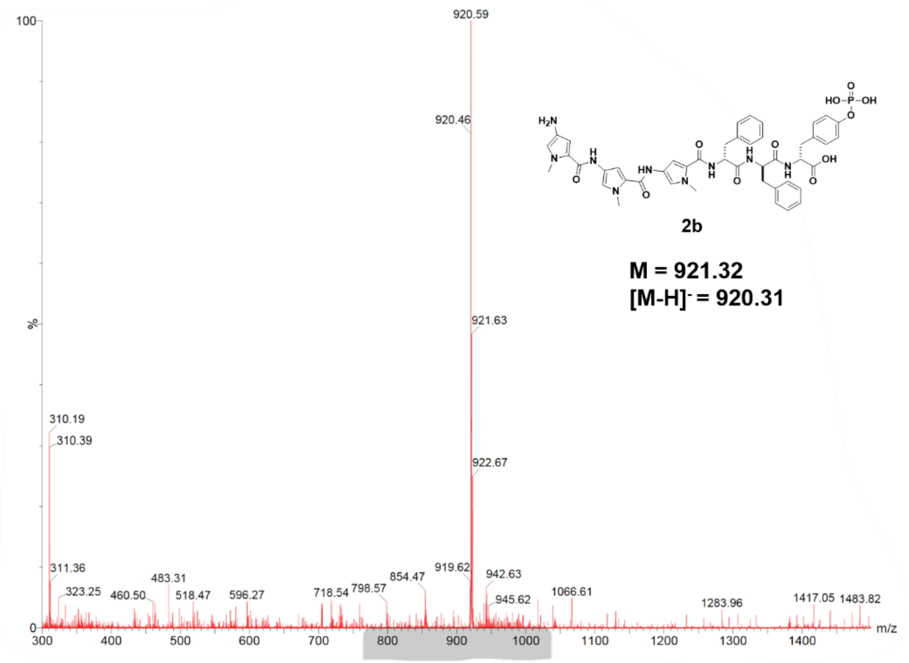

Figure S4. Mass spectrum of **2b** (*m/z* = 920.59).

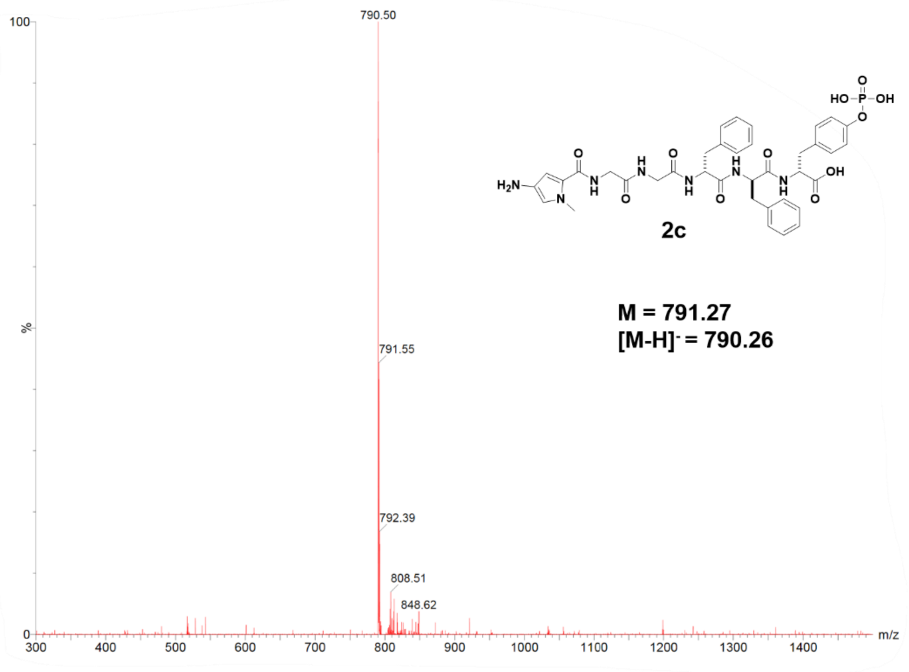

Figure S5. Mass spectrum of **2c** (*m/z* = 790.50).

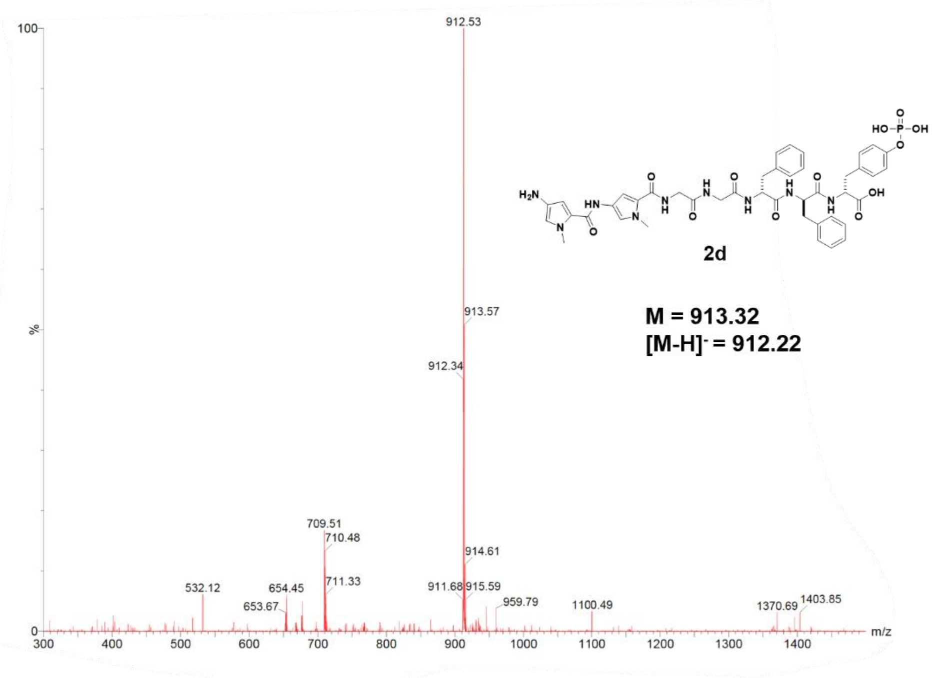

Figure S6. Mass spectrum of **2d** (m/z = 912.53).

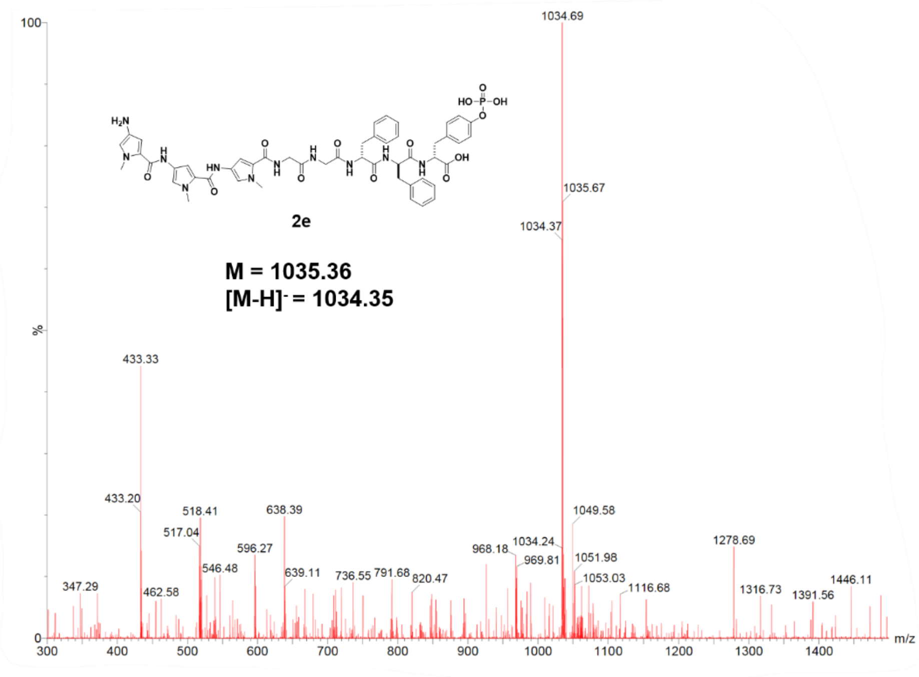

Figure S7. Mass spectrum of **2e** (m/z = 1034.69).

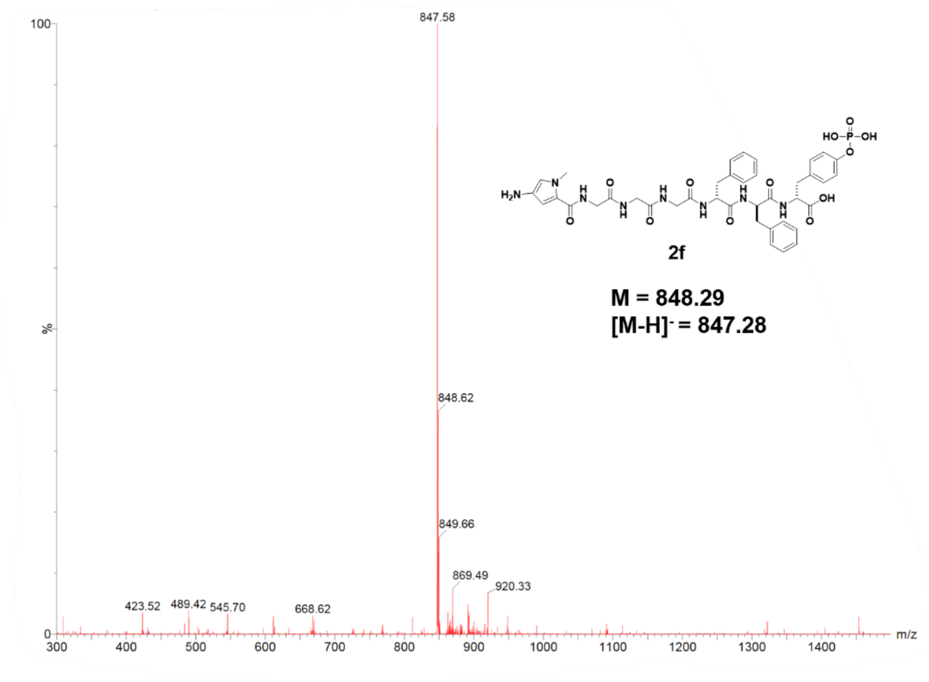

Figure S8. Mass spectrum of **2f** (*m/z* = 847.58).

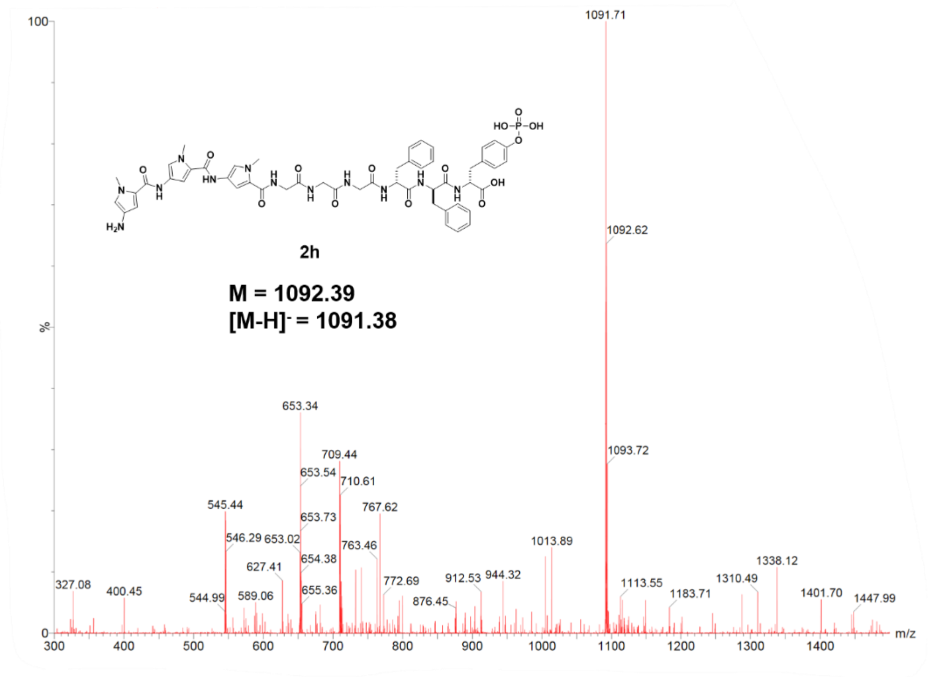

Figure S9. Mass spectrum of **2h** (*m/z* = 1091.71).

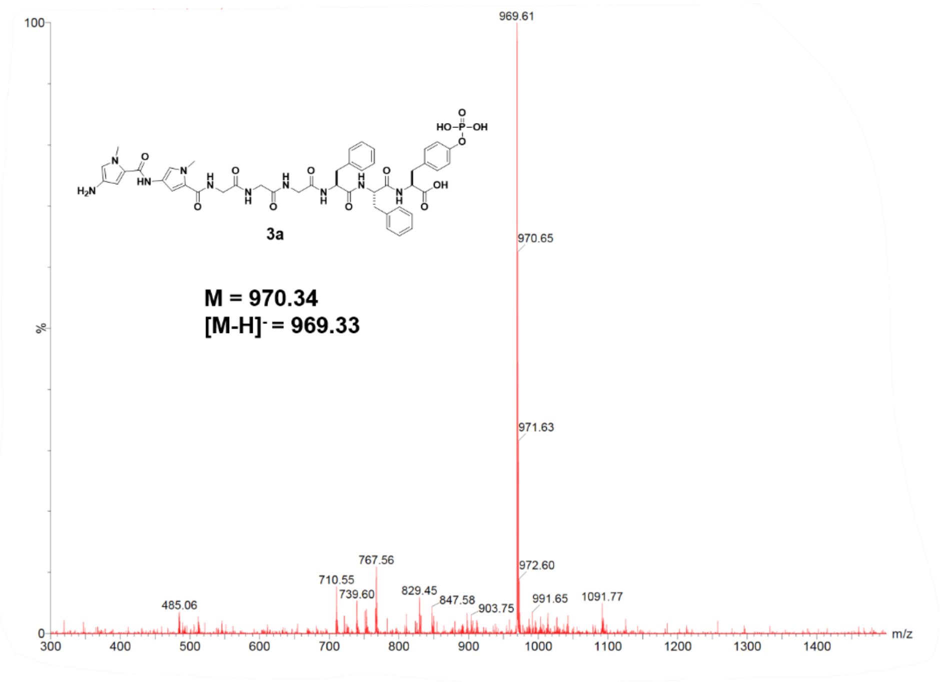

Figure S10. Mass spectrum of **3a** (*m/z* = 969.61).

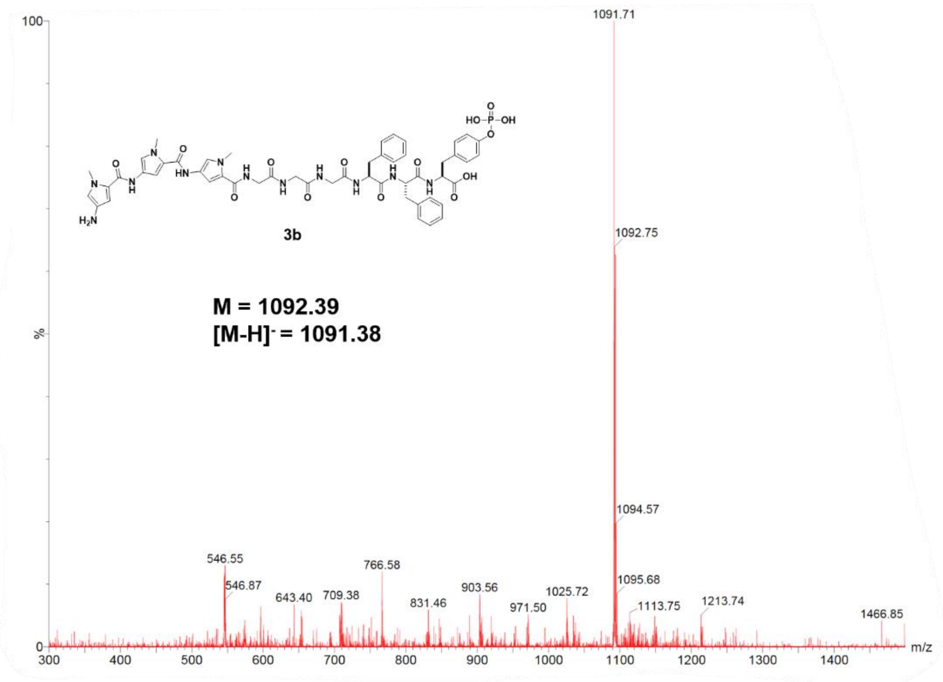

Figure S11. Mass spectrum of **3b** (*m/z* = 1091.71).

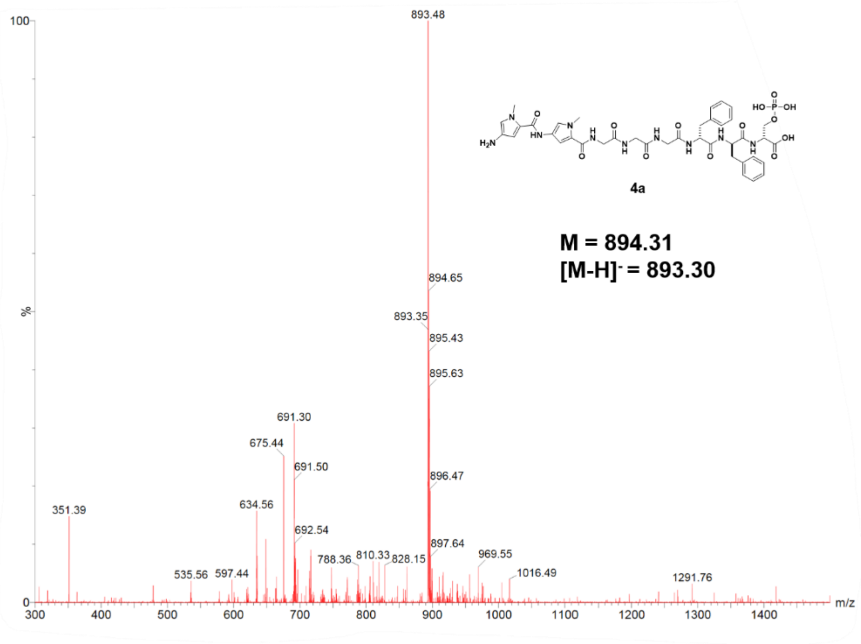

Figure S12. Mass spectrum of **4a** (*m/z* = 893.48).

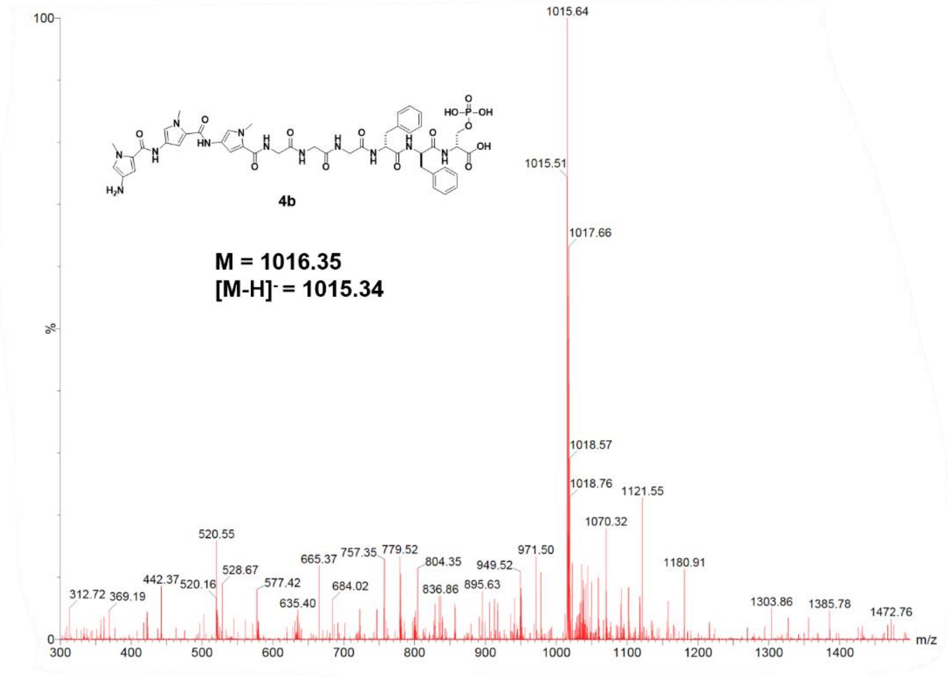

Figure S13. Mass spectrum of **4b** (*m/z* = 1015.64).

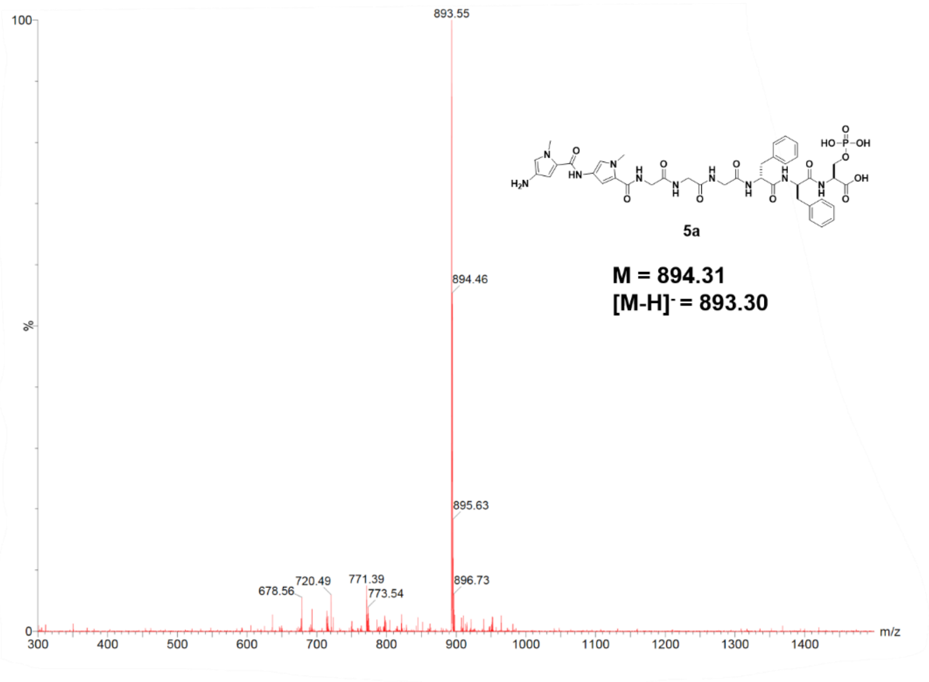

Figure S14. Mass spectrum of **5a** (*m/z* = 893.55).

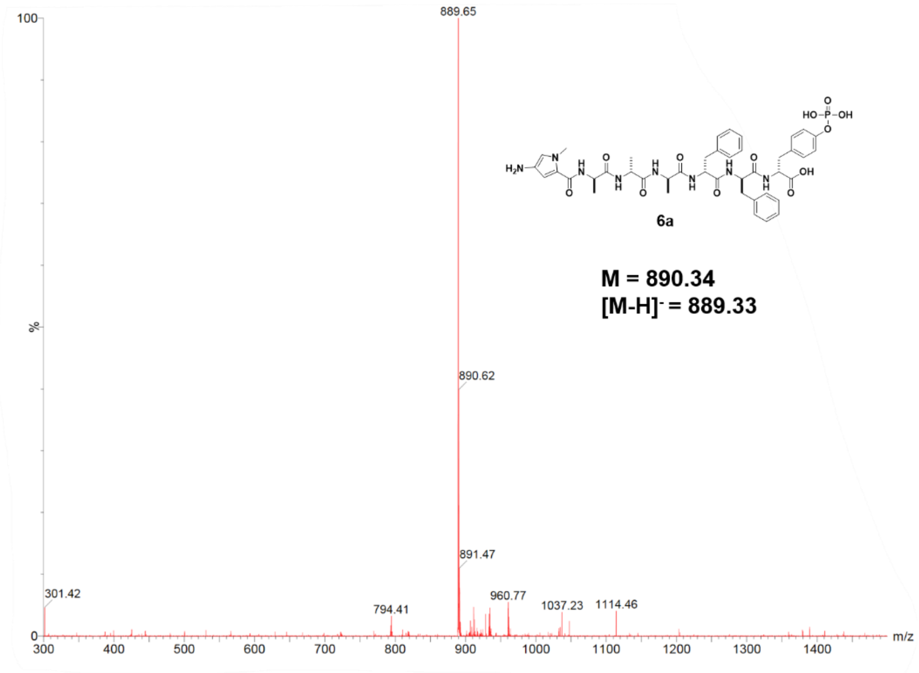

Figure S15. Mass spectrum of **6a** (*m/z* = 889.65).

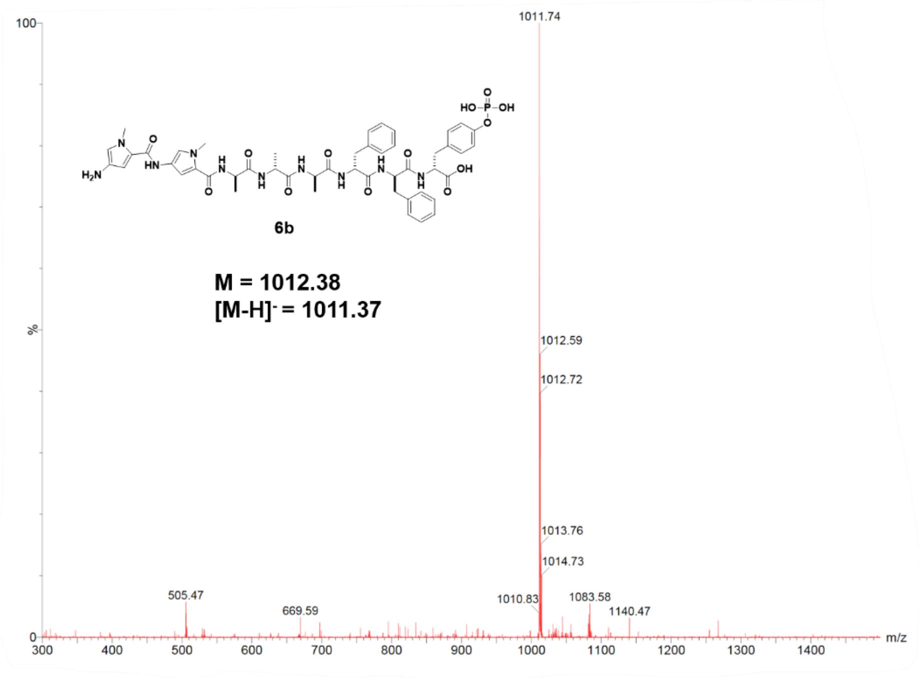

Figure S16. Mass spectrum of **6b** (*m/z* = 1011.74).

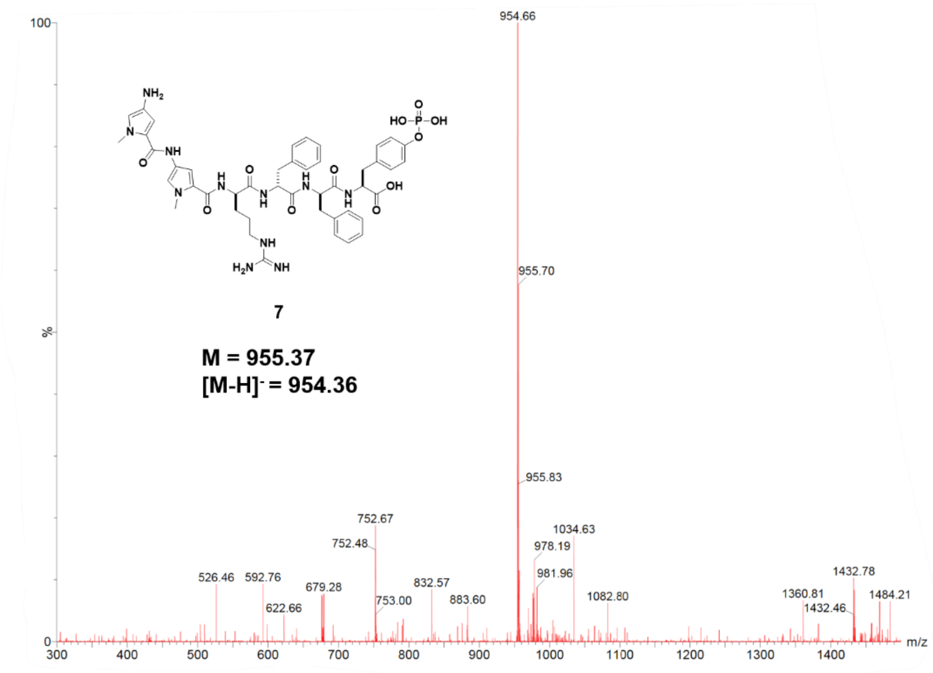

Figure S17. Mass spectrum of **7** (*m/z* = 954.66).

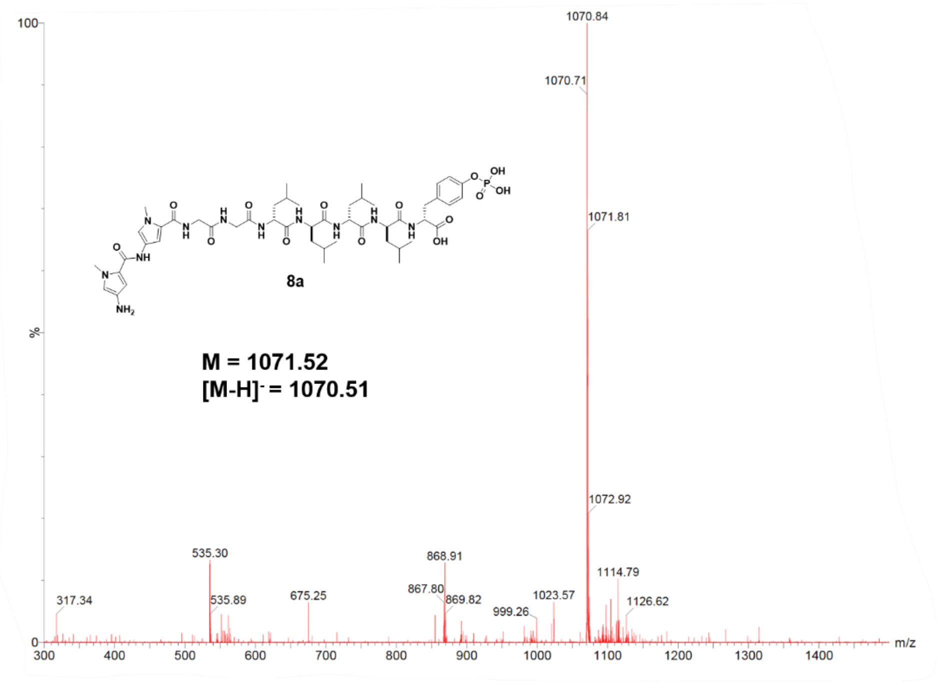

Figure S18. Mass spectrum of **8a** (*m/z* = 1070.84).

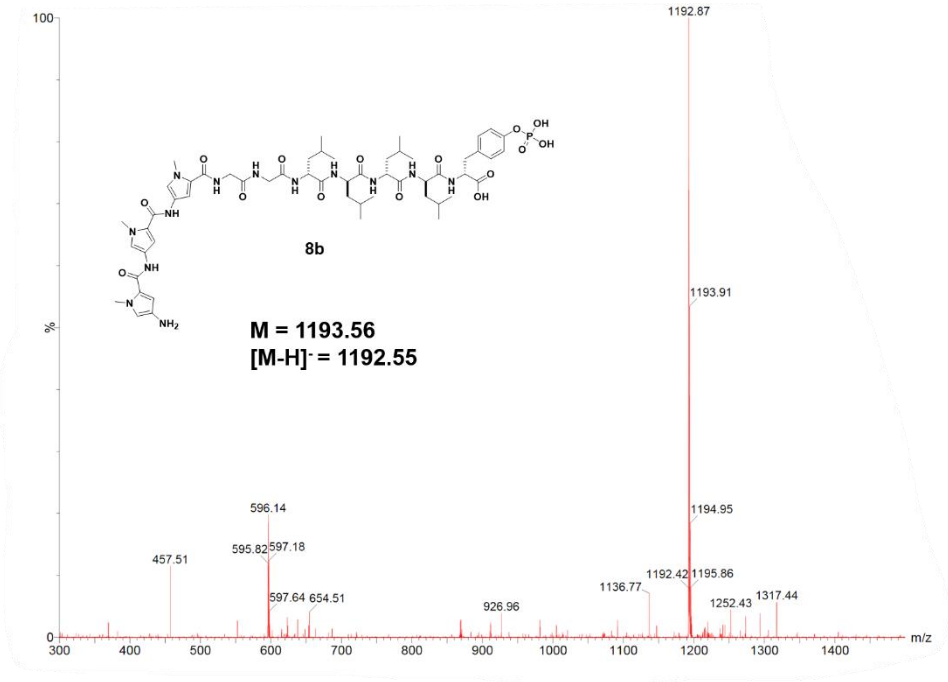

Figure S19. Mass spectrum of **8b** (*m/z* = 1192.87).

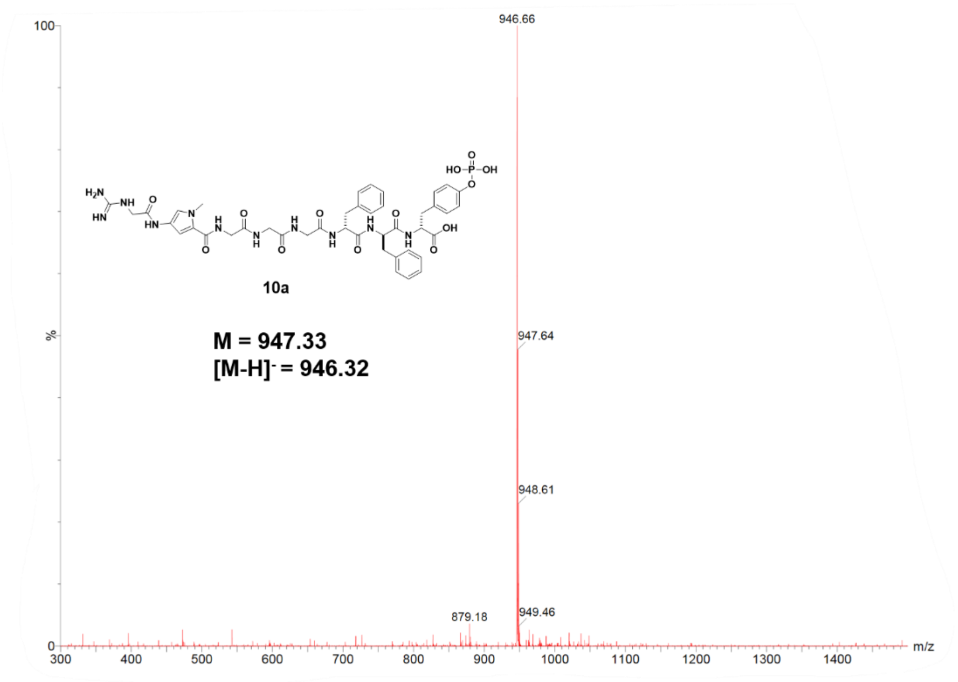

Figure S20. Mass spectrum of **10a** (*m/z* = 946.66).

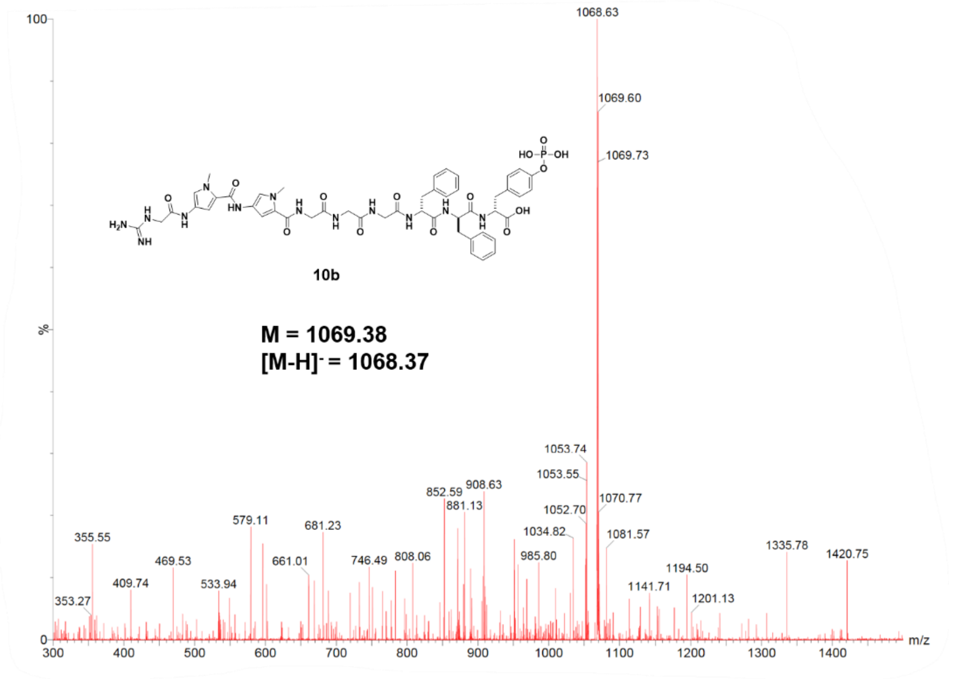

Figure S21. Mass spectrum of **10b** (*m/z* = 1068.63).

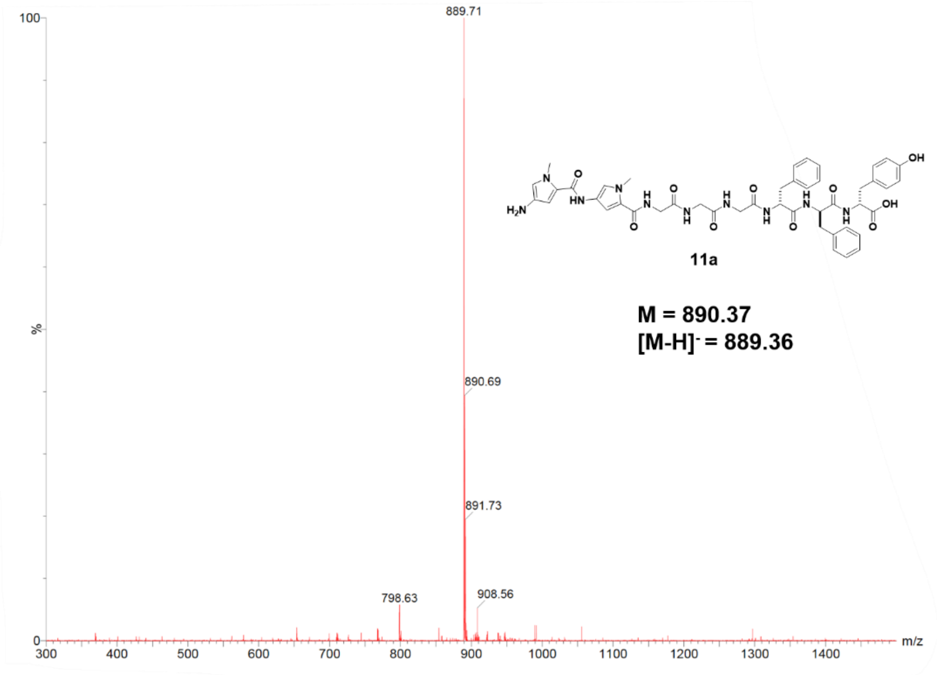

Figure S22. Mass spectrum of **11a** (*m/z* = 889.71).

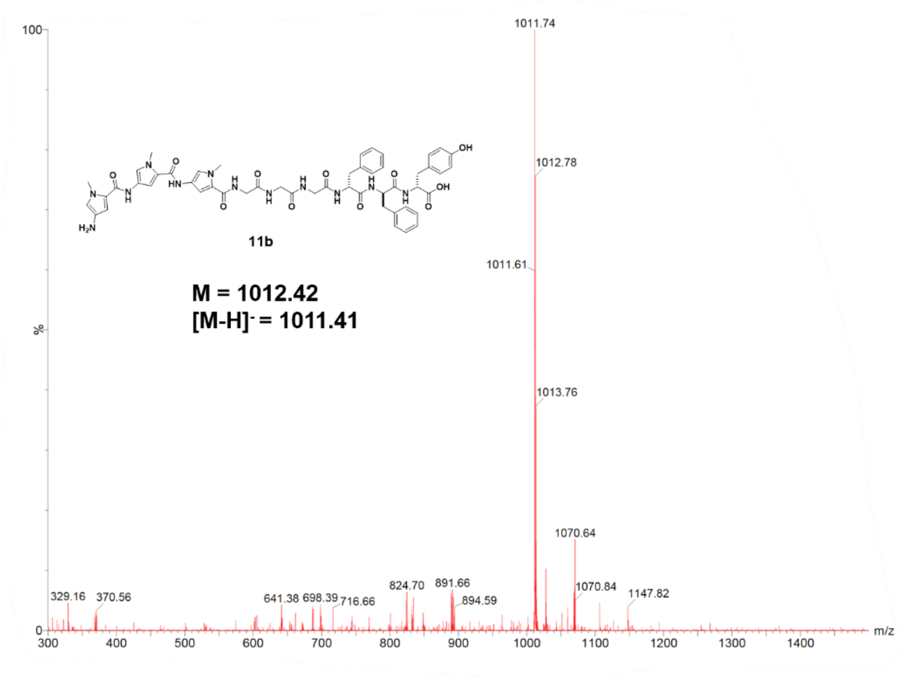

Figure S23. Mass spectrum of **11b** (*m/z* = 1011.74).

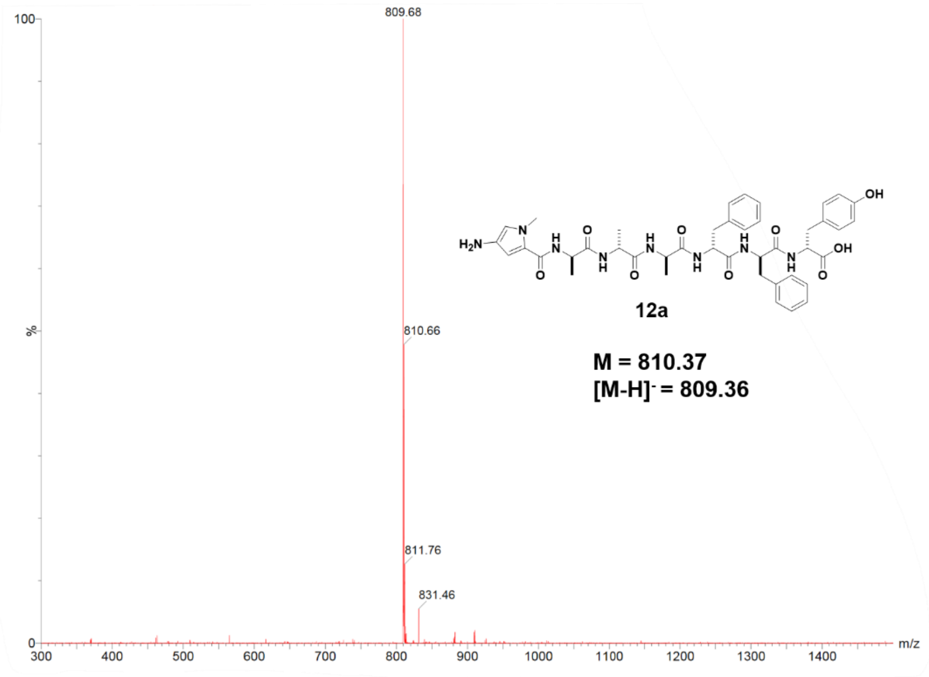

Figure S24. Mass spectrum of **12a** (*m/z* = 809.68).

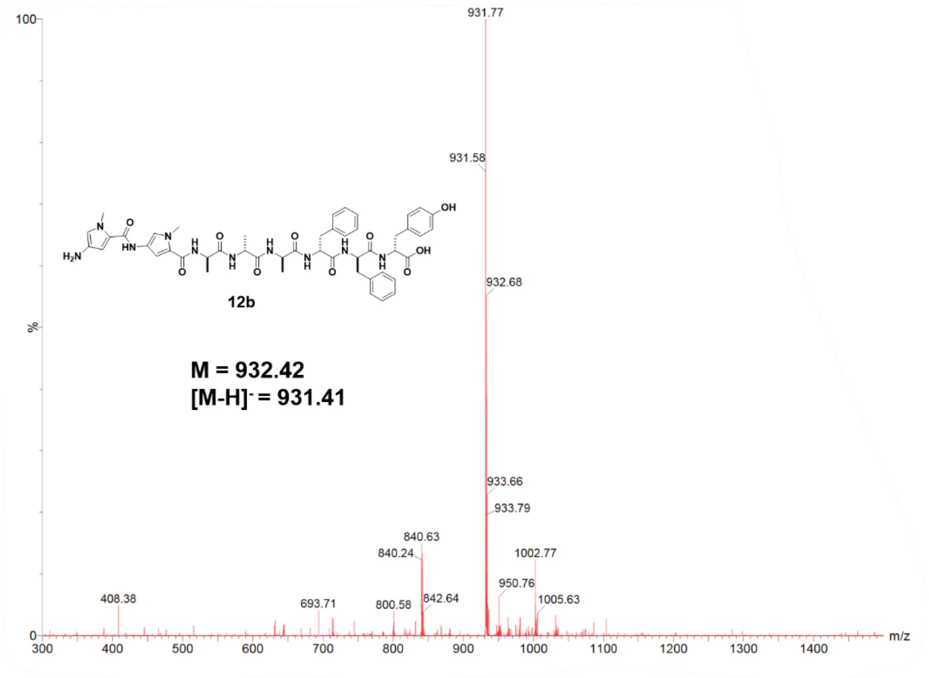

Figure S25. Mass spectrum of **12b** (*m/z* = 931.77).

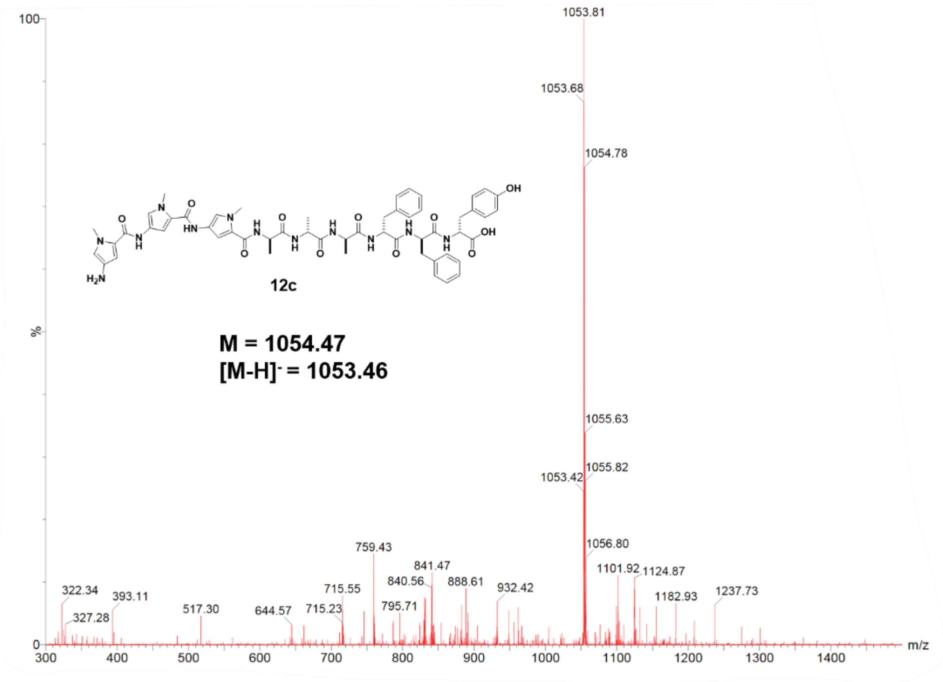

Figure S26. Mass spectrum of **12c** (*m/z* = 1053.81).

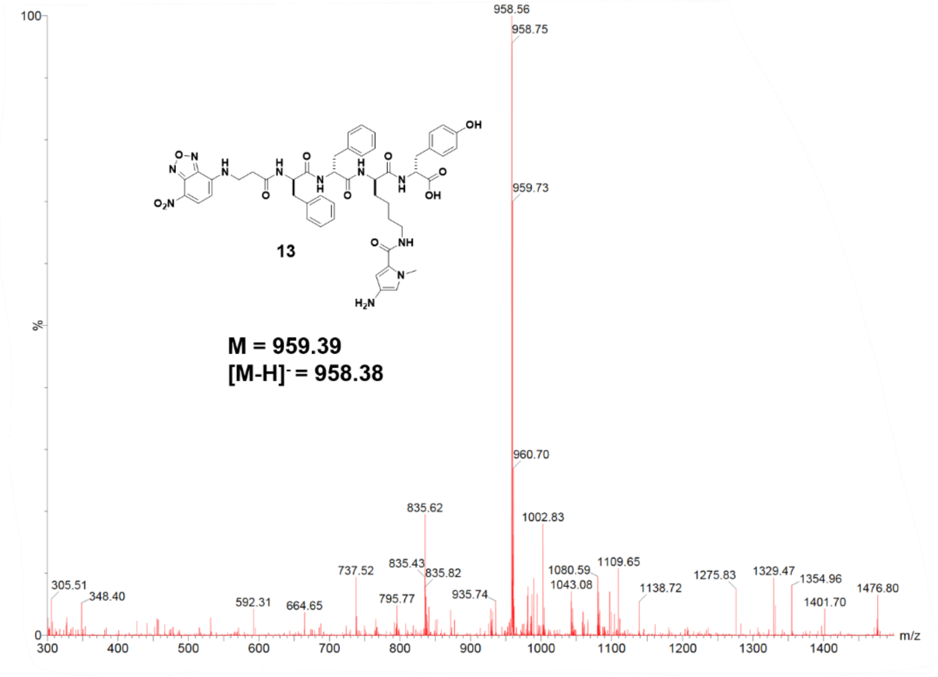

Figure S27. Mass spectrum of **13** (*m/z* = 958.56).

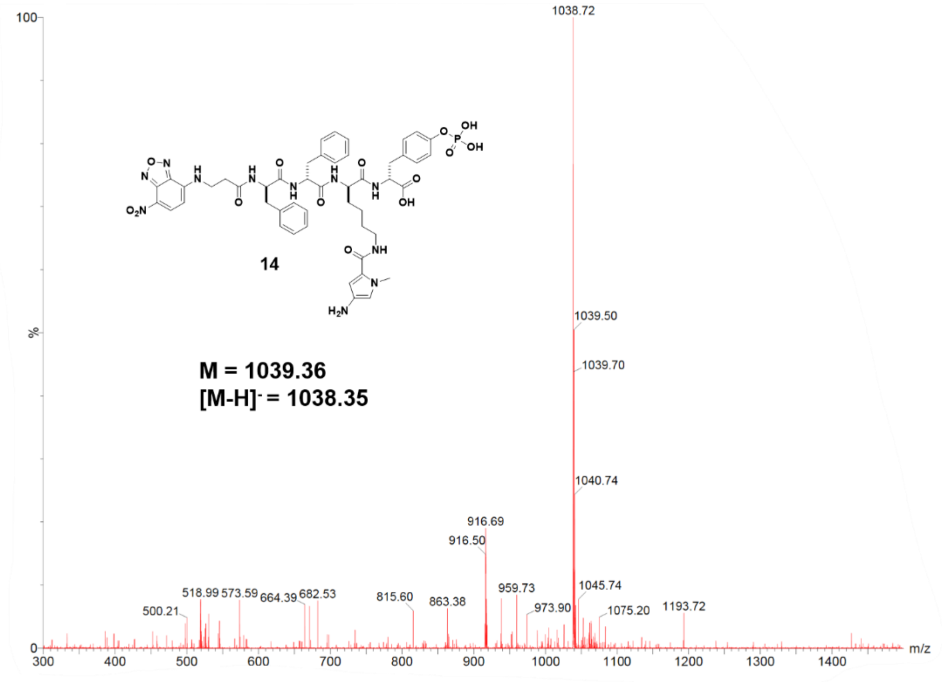

Figure S28. Mass spectrum of **14** (*m/z* = 1038.72).

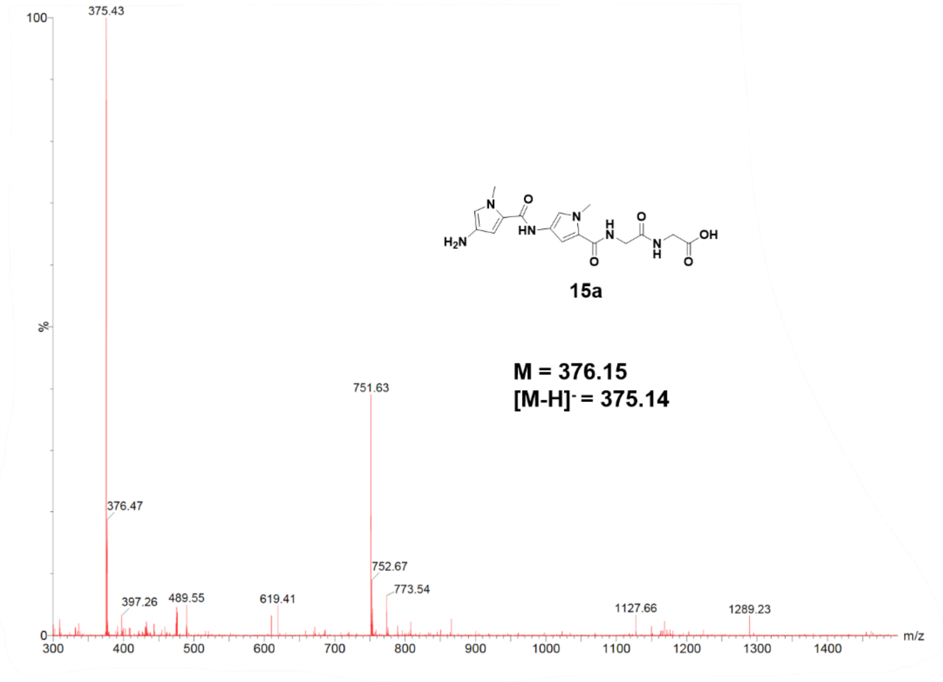

Figure S29. Mass spectrum of **15a** (*m/z* = 375.43).

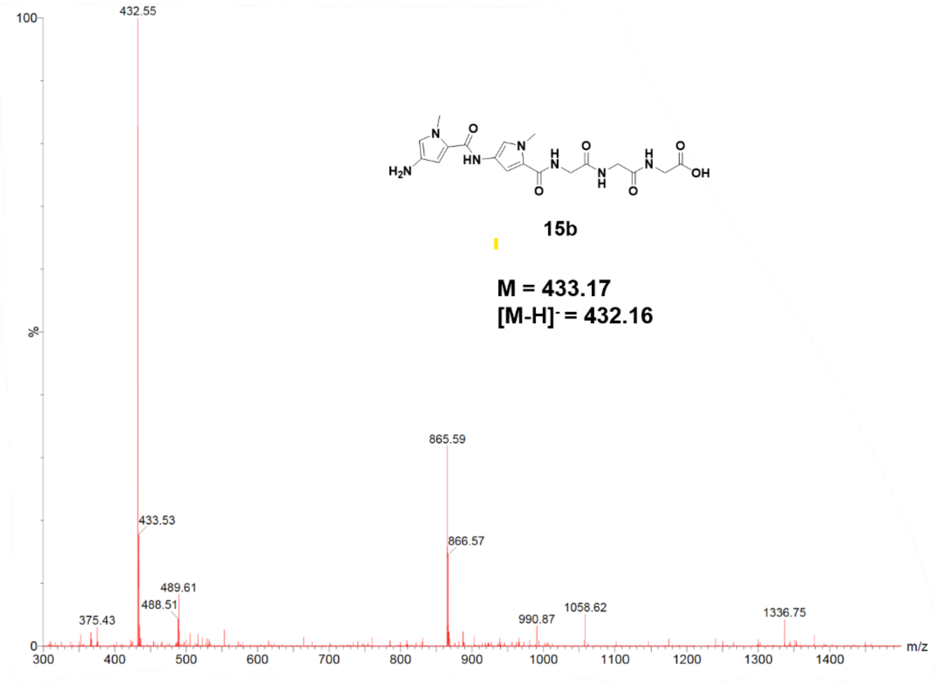

Figure S30. Mass spectrum of **15b** (*m/z* = 432.55).

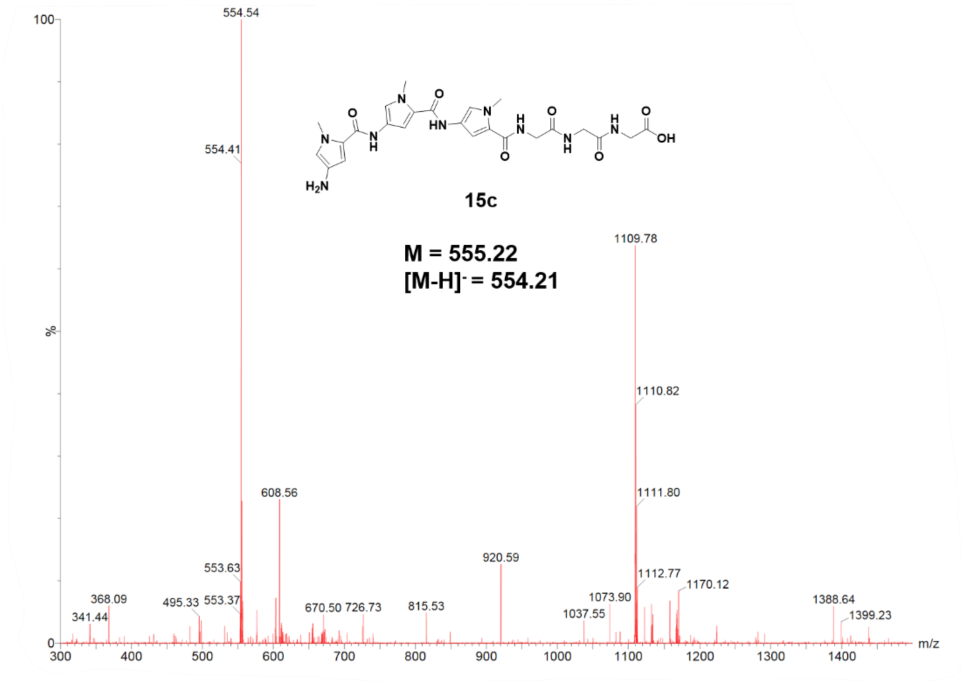

Figure S31. Mass spectrum of **15c** (*m/z* s= 554.54).

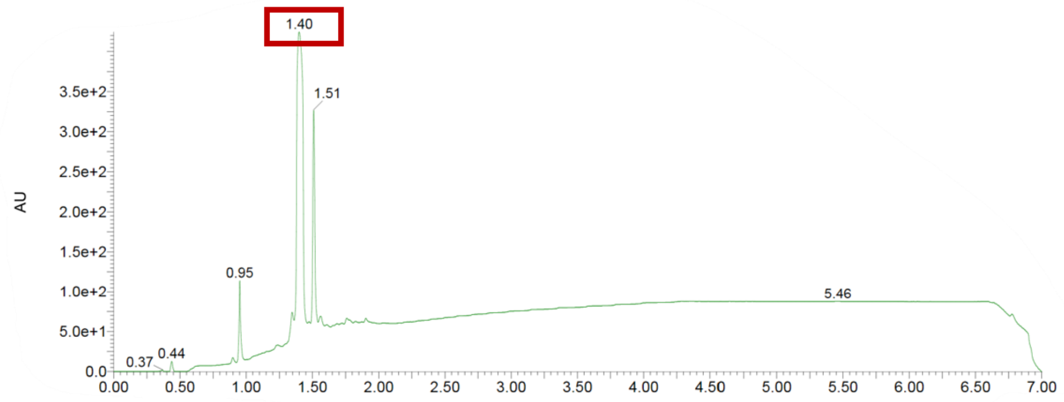

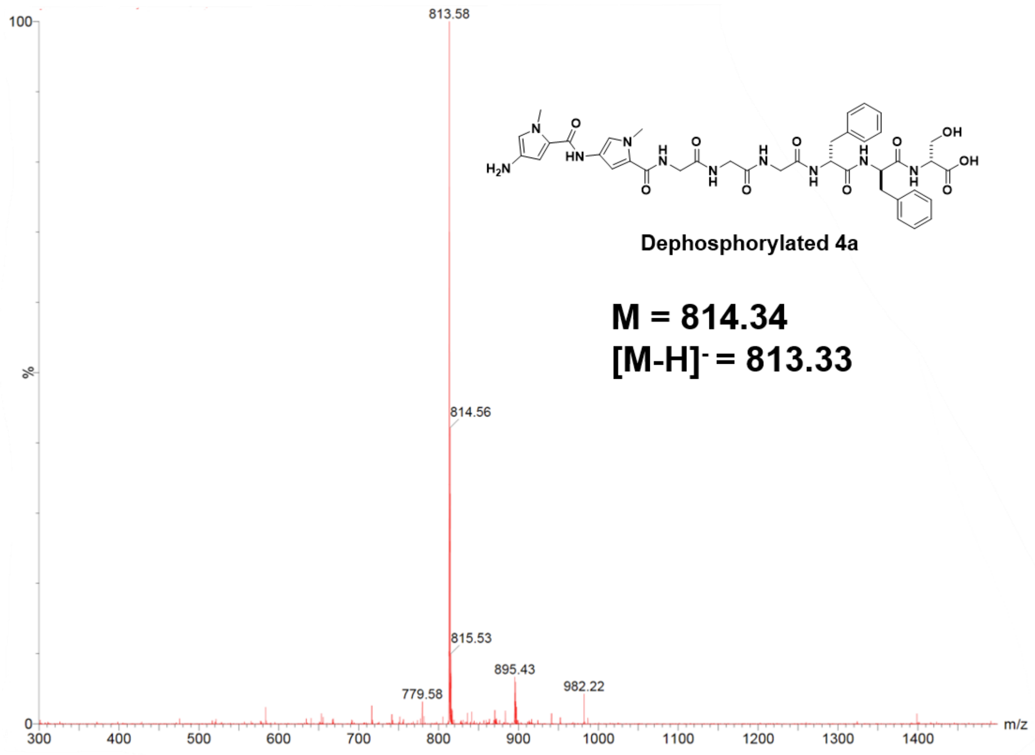

Figure S32. LC spectrum and mass spectrum of dephosphorylated **4a** (treated with 1 U/mL ALP for 24 h).

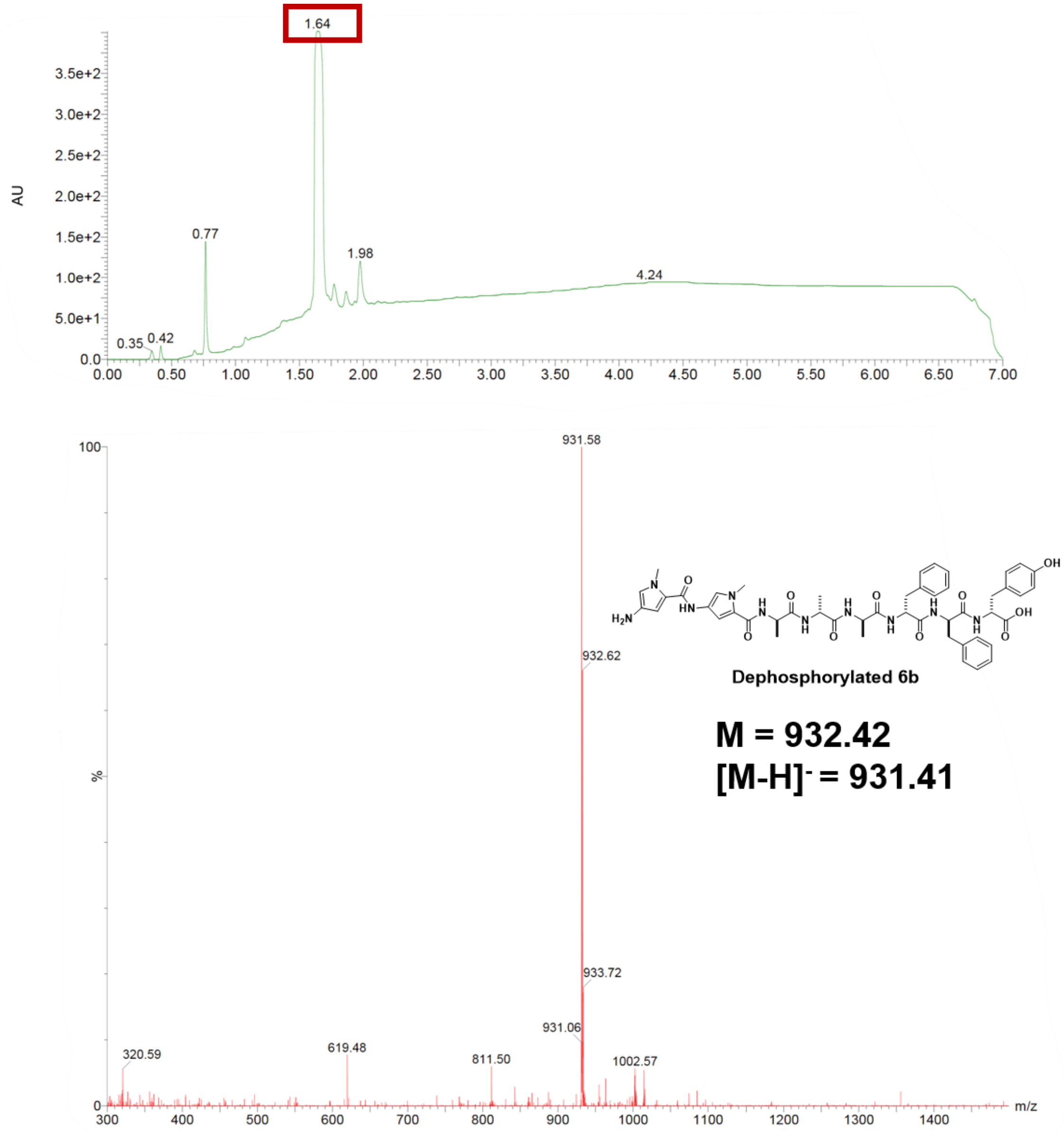

Figure S33. LC spectrum and mass spectrum of dephosphorylated **6b** (treated with 1 U/mL ALP for 24 h).

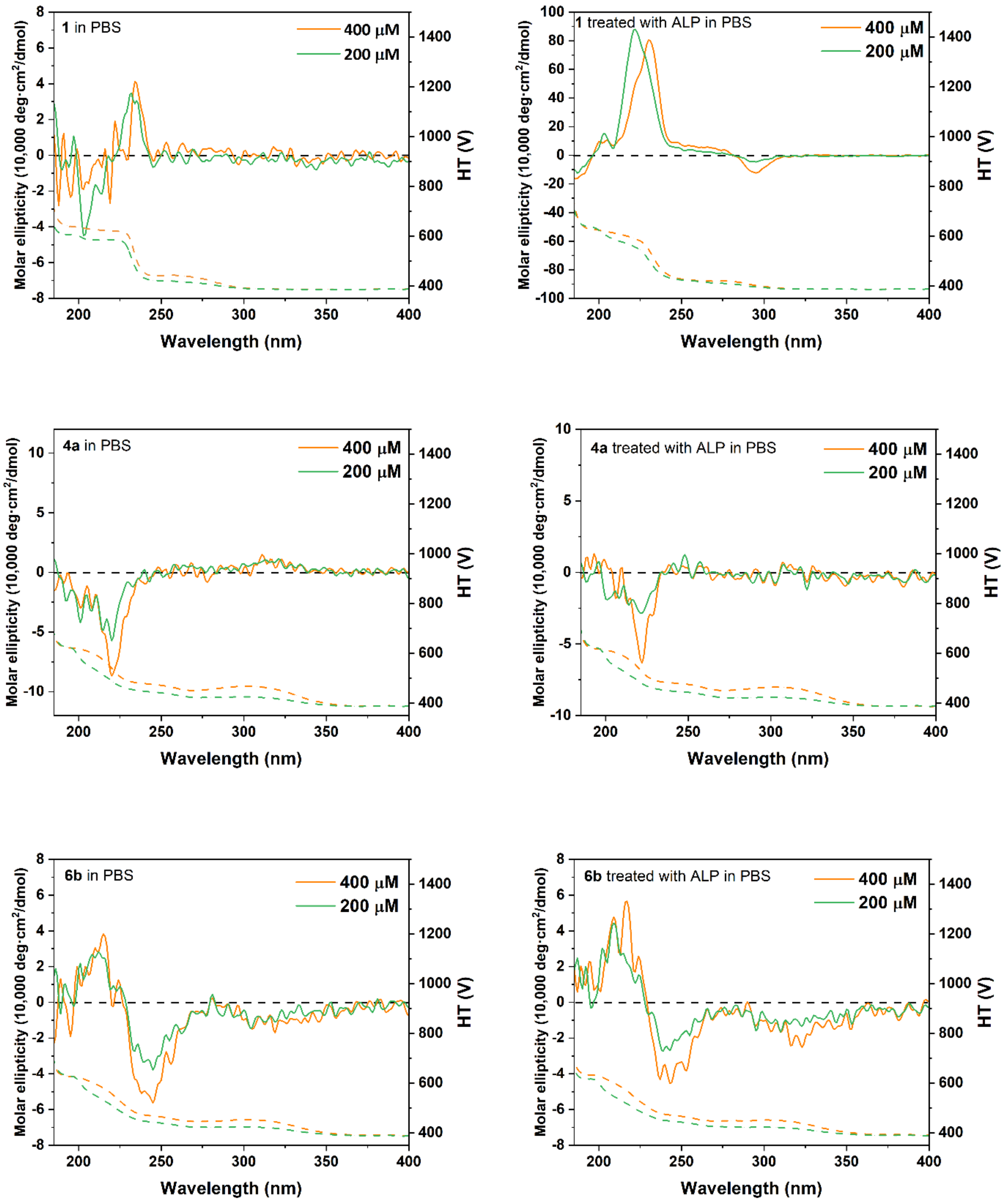

Figure S34. CD spectra of **1**, **4a** and **6b** before and after treatment with 1 U/mL ALP for 24 h.

1. Handbook, M. B., ; Walker, JM, Rapley, R., Eds. Humana Press: Totowa, NJ: 2008.
